# Inter-cofactor protein remodeling rewires short-circuited transmembrane electron transfer

**DOI:** 10.1038/s42004-025-01460-y

**Published:** 2025-04-09

**Authors:** Deborah K. Hanson, James C. Buhrmaster, Ryan M. Wyllie, Gregory A. Tira, Kaitlyn M. Faries, Dewey Holten, Christine Kirmaier, Philip D. Laible

**Affiliations:** 1 Biosciences Division, Argonne National Laboratory, Lemont, IL USA; 2 Department of Chemistry, Washington University, St. Louis, MO USA

**Keywords:** Biophysical chemistry, Structural biology, Proteins

## Abstract

Intraprotein electron transfer (ET) requires explicit local control of the environment of cofactors to influence their intermolecular distances, relative orientations, and redox properties. Efficient, longer-range ET often utilizes molecular orbitals of aromatic residues present in the intervening space. Here, revitalization of a vestigial ET pathway in the bacterial photosynthetic reaction center is achieved by scanning with tryptophans to uncover markedly improved routes of electron conduction in a key stabilizing step spanning 15 Å between tetrapyrrole and quinone cofactors. This ET event is maximally enhanced by pairing one or more tryptophans with a threonine to influence quinone binding and/or redox potential. Synergistic effects of these substitutions increase the yield of that ET step to ~95%. Joining these substitutions with mutant residues that improve initial ET steps dramatically enhances transmembrane charge separation via this redesigned version of a pathway that is quantitatively inactive in the native protein-cofactor complex.

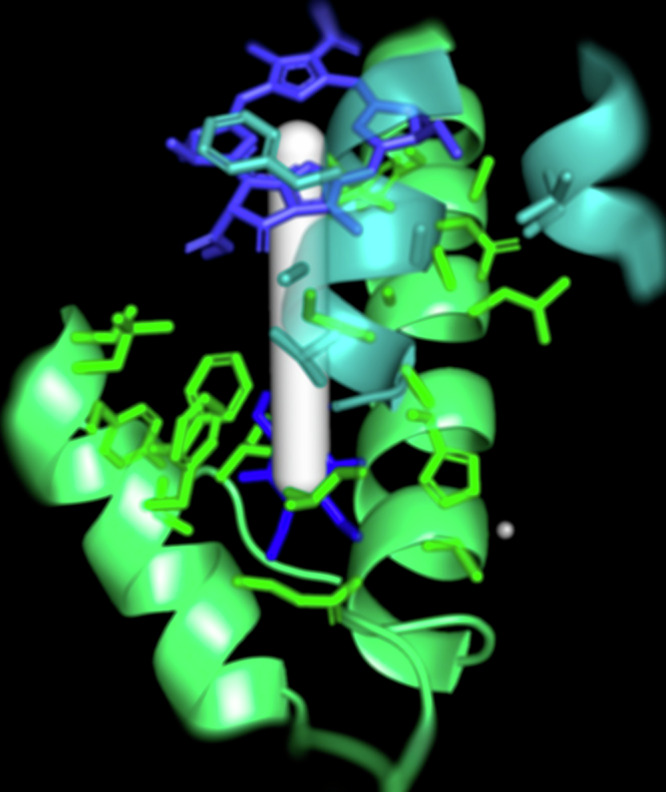

## Abstract (web summary)

Light-driven electron transfer (ET) in bacterial photosynthetic reaction centers proceeds in a transmembrane protein complex with two symmetric pathways of cofactors – the A pathway operational, while the B pathway is effectively inoperative – leaving efficient activation of the B pathway a challenge. Here, the authors redesign the non-functional B branch in the *Cereibacter sphaeroides* photosynthetic reaction center, enhancing long-range ET by incorporating tryptophan residues at strategic positions between cofactors, achieving variants with impressive transmembrane charge separation yields due to the increased competency of secondary ET (up to 95%).

## Introduction

Efficient, protein-mediated electron transfer (ET) reactions are essential for the life of any cell. Protein-cofactor interactions are critical for binding and positioning the electron carriers for maximum electronic coupling and for tuning their properties to ensure productive movement of charges. When cofactors are positioned at distances that preclude orbital overlap, efficient long-range ET requires electron-rich channels to increase electronic coupling between cofactors. Aromatic residues often assume these roles. They can accelerate ET reactions by acting as discreet redox intermediates^1–3^ or by bridging two cofactors to mediate superexchange^4–6^ via frontier molecular orbitals that are of similar energy. They can also stabilize nearby charge-separated states or act in a protective role to divert excess electrons or excited states^1^.

**Fig. 1:**
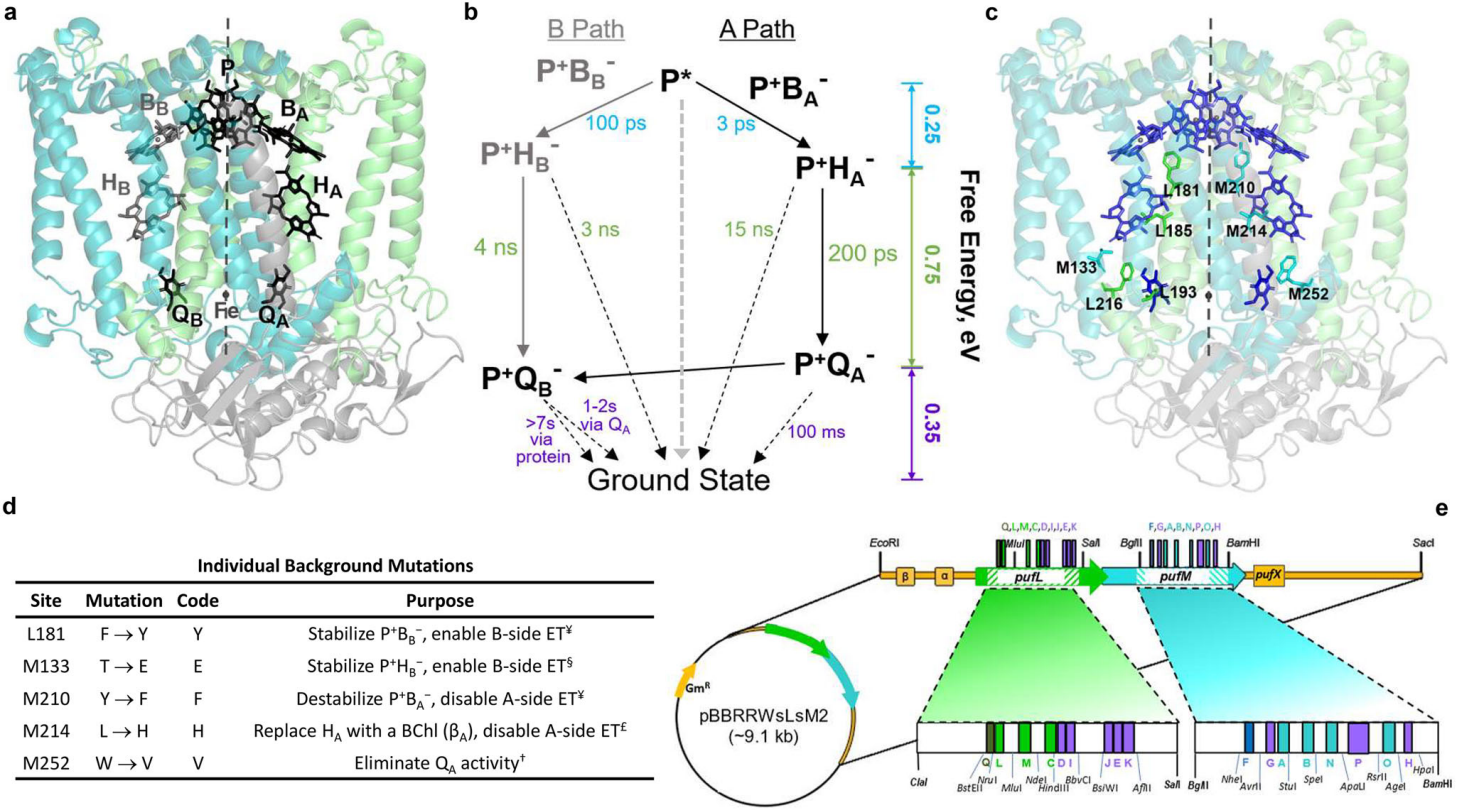
Manipulation of ET on the A and B cofactor pathways. The structure of the *C. sphaeroides* reaction center (**a**, PDB code: 1aig). Three subunits – L (green), M (cyan), and H (gray) – bind cofactors as described in the text. An axis of pseudo-C_2_ symmetry (dashed line) relates the cofactors and the homologous transmembrane L and M chains. Phytyl tails of cofactors and the carotenoid were removed for visual clarity. **b** Free energy diagram for the native RC showing intermediates and time constants for both A (black) and B (gray) ET (solid lines), and CR (dashed) pathways. **c** Structural positions of the native residues of the L and M chains that are substituted herein to result in high yields of B-side ET to form P^+^Q_B_^─^. **d** Functional consequences of background mutations in the parent strain, YEFHV. ^¥^Calculations^19,21,65^ indicate that TyrM210 stabilizes P^+^B_B_^−^ by ~200 meV whereas the symmetry-related PheL181 does not provide such stabilization of P^+^B_B_^−^. ^§^A hydrogen bond between GluM133 and the ring V keto group of H_B_ red shifts the Q_x_ band of H_B_ from 528 to 535 nm^28^ and should stabilize P^+^H_B_^−^ by 50−100 meV based on the effects of hydrogen bonds to bacteriochlorophyll *a* and bacteriopheophytin *a*.^66,67^^£^The cofactor substitution^32,68–71^ spectrally isolates the Q_x_ band, enabling unambiguous observation and analysis of P^+^H_B_^−^. P^+^β_A_^−^ is expected to lie higher in free energy than P^+^H_A_^−^ by 150−300 meV based on the difference in reduction potentials of BChl *a* and BPh *a* in vitro.^72,73^^†^The W(M252)V^74^ substitution weakens binding of Q_A_ resulting in its displacement or absence. No Q_A_ is present in the X-ray crystal structure of the W(M252)V (PDB code 7mha)^75^ *C. sphaeroides* mutant RC. **e** Engineered expression vector for high-throughput mutagenesis of the *C. sphaeroides* RC^28^. Unique restriction sites flank segments of the L and M genes targeted for mutagenesis (striped regions), facilitating directed insertions of synthetic oligonucleotide cassettes encoding the desired substitutions. A unique, silent *Bst*EII site was added to expression plasmid pBBRRWsLsM^28^ to delineate segment Q, flanked on the 3’ end by *Nru*I. Substitutions targeted in this study are located within regions designated by additional colors (dark green, Phe cluster; purple, Trp scanning set; dark cyan, Trp cluster).

The importance of protein-cofactor interactions in directing the first steps in light-driven transmembrane ET in photosynthetic organisms is illustrated exquisitely in the reaction center (RC). The RC from the purple non-sulfur bacterium *Cereibacter* (*C*.; formerly *Rhodobacter*^7^) *sphaeroides* consists of three protein subunits – L, M, and H – that bind a dimer of bacteriochlorophylls that serves as the primary electron donor (P), two monomeric bacteriochlorophylls (B), two bacteriopheophytins (H), two ubiquinone_10_ molecules (Q), a non-heme iron atom, and a carotenoid. The crystal structure of the RC^8–10^ shows that the five-helix, integral membrane L and M subunits form a heterodimer in which the axis of approximate C_2_ symmetry relating the subunits also applies to their associated cofactor branches (Fig. 1a). The first three cofactors in the ET pathway of either branch – P, B_A or B_, and H_A or B_ – are tetrapyrroles that are bound by the protein in close proximity with edge-to-edge distances of ~5 Å between the highly-conjugated macrocycles. On each branch, a greater physical distance ( ~ 10 Å edge-to-edge, ~14 Å center-to-center) separates the bacteriopheophytin cofactor from the quinone.

Over a time span of more than three billion years, an ancestral homodimeric RC has diverged such that ET in the bacterial RC proceeds solely via the A pathway (Fig. 1a, b) while the B pathway is effectively inoperative (reviewed in Ref. ^11^). When a photon excites P, ET proceeds from the periplasmic side to the cytoplasmic side of the membrane in a stepwise fashion from P* (excited state of P) to A-branch cofactors B_A_, H_A_, and Q_A_, then to the terminal acceptor Q_B_ (Fig. 1b). Following a second excitation of P, doubly-reduced Q_B_ acquires two protons, Q_B_H_2_ diffuses from the RC to deliver electrons to the cytochrome *bc*_1_ complex, and P^+^ is reduced twice by periplasmic cytochrome *c*_2_ to complete the 2e^–^/2H^+^ cycle.

Electron flow is optimized to near unity quantum yield for all steps along the A pathway while the B side has evolved to stabilize/sequester the semiquinone intermediate in the Q_B_ binding pocket prior to the second ET event^11–13^. The initial (“primary”) steps of ET – P* → B_A_ → H_A_ – are rapid as the free energies of charge-separated states involving these cofactors are closely spaced and energetically downhill from each other (Fig. 1b;^14^). “Secondary” ET from H_A_ to Q_A_ occurs with a quantum yield of near unity across the ~10 Å distance at a slower rate and a greater drop in free energy. ET from Q_A_ to Q_B_ is an even slower gated process^15,16^ that occurs on the microsecond timescale^17^ (Fig. 1b). Although both quinones are UQ_10_ molecules in the *C. sphaeroides* RC, their functional properties differ in that Q_A_ is a tightly-bound, single-electron acceptor that is never protonated.

The L and M subunits position the identical A- and B-side cofactors on similar scaffolds where there are a number of local amino-acid asymmetries – many of which are highly conserved in related organisms – that affect the microenvironments of the cofactors (Fig. 1c). Protein-influenced differences in the physical and functional properties of the cofactors determine their ground state spectra, electronic coupling, and the free energies of the respective charge-separated states on the highly-optimized A pathway versus the non-functional B pathway.

Dating to the initial discovery of the RC structure, activation of the normally inoperative B pathway via directed mutagenesis has been the objective of many studies (reviewed in Ref. ^18^) that (i) target elimination or reversal of conserved, amino-acid asymmetries, (ii) impair the A pathway, and/or (iii) enable the B pathway. These strategies must consider the substantial differences in the rates of both the ET and charge recombination (CR) reactions on the two pathways in the wild-type (WT) RC (Fig. 1b). The rates of primary ET to form the P^+^H^–^ state on either pathway of the native RC are ~30× greater on the A side where the balance between ET and CR greatly favors ET and accumulation of P^+^H_A_^–^. The corresponding rates of secondary ET from H to Q also differ on the two paths by more than an order of magnitude in RCs with native residues surrounding H_A_/H_B_ and Q_A_/Q_B_. The high quantum yield of P^+^Q_A_^–^ that is obtained from photochemistry using the A-side cofactors is due in large part to the fact that P^+^H_A_^–^ → P^+^Q_A_^–^ ET is approximately two orders of magnitude faster than CR of the P^+^H_A_^–^ state (Fig. 1b; time constants of 200 ps vs. 15 ns). In contrast, on the native B pathway the free energy of the P^+^B_B_^–^ state is higher than that of P* (Fig. 1b)^19–26^. The slow formation of P^+^H_B_^–^ in ~100 ps is coupled with a secondary ET step where formation of P^+^Q_B_^–^ from P^+^H_B_^–^ is disfavored over CR of P^+^H_B_^–^ to the ground state (Fig. 1b; ~ 4 ns vs. 3 ns, respectively^27^).

A semi-directed mutagenesis strategy (e.g., Fig. 1d) was used previously to construct mutant RCs of related organisms *Rhodobacter* (*R*.) *capsulatus* and *C. sphaeroides* in which ET to the A-side cofactors is severely disabled (reduced to ~10–30% of native RC) and a significant portion of primary ET from P* is redirected to the normally inactive B-path cofactors, producing an ~70–90% yield of P^+^H_B_^–^ ^27–34^. However, the quantum yield of the subsequent secondary P^+^H_B_^–^ → P^+^Q_B_^–^ ET step in those RCs remains poor, ranging from ~12 to 40%. The path forward towards a stablized, transmembrane charge-separated state is short-circuited at this step by the unfavorable balance between secondary P^+^H_B_^–^ → P^+^Q_B_^–^ ET and CR of P^+^H_B_^–^ (Fig. 1b). This characteristic of the B-side ET pathway remains a formidable barrier that must be overcome to achieve quantitative B-path reduction of Q_B_.

On the highly-optimized A pathway, the (200 ps)^–1^ rate for the P^+^H_A_^–^ → P^+^Q_A_^–^ secondary ET step is rapid with respect to the 10 Å distance separating H_A_ and Q_A_. Long-range ET between these cofactors is mediated (at least in part) by electronic coupling involving a conserved tryptophan residue that is in van der Waals contact with both H_A_ and Q_A_^8,35^. These interactions are largely missing between H_B_ and Q_B_ on the B pathway. This conserved asymmetry and other dissimilarities in the aromatic residues that surround the bacteriopheophytin cofactors (H_A_ or H_B_) may be principal components of the differences in functionality of the A and B pathways in secondary ET to produce and stabilize reduced quinones^6,36–38^.

This striking, nature-designed inclusion of a strategically positioned and carefully oriented tryptophan between H_A_ and Q_A_ in the native RC complex initiated a multifaceted search—with parallel directed and scouting-type approaches—for a means to achieve the same yield of secondary ET on the B-side pathway. In this endeavor, differences in aromatic residues in the environments of both bacteriopheophytin acceptors were explored, electronic coupling between H_B_ and Q_B_ was enhanced by beneficial placement of tryptophan residues, and rational engineering was used to optimize tryptophan orientation and quinone binding. Remodeling of the region between H_B_ and Q_B_ resulted in the ultimate realization of RC variants characterized by near-quantitative yields of secondary ET on the B path.

## Results

The “YEFHV” RC (Fig. 1d;^28,32,33^) served as the parent to which other substitutions were added in this study with the aim of improving the yield of the secondary P^+^H_B_^–^ → P^+^Q_B_^–^ ET step. Primary ET in this RC is characterized by P* decay of 72% to the B side, 12% to the A side (ET stops with formation of P^+^β_A_^−^), and 16% to internal conversion to the ground state^32,33^. The yield of the secondary ET step is only ~40%, resulting in an overall yield of multi-step B-side ET to Q_B_ of 28%.

### Differential electron density in the environments of H_A_ and H_B_

**Fig. 2:**
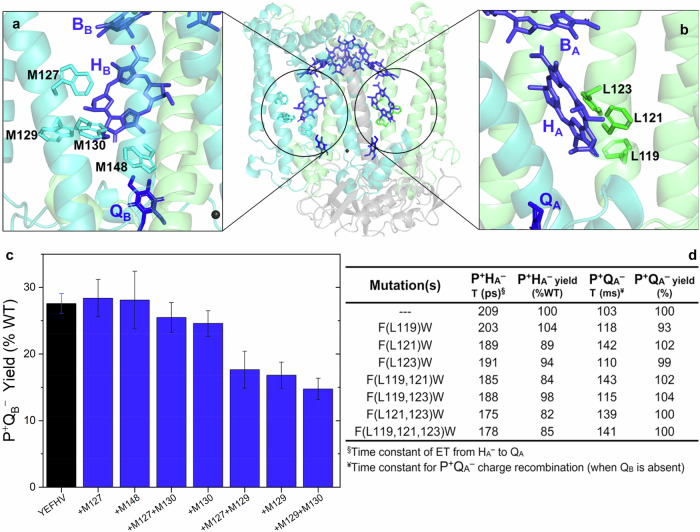
Consequences of alterations within clusters of aromatic amino acids near bacteriopheophytins. Sites of residues of the Trp cluster near H_B_ that were substituted singly and in combination with Phe (**a**, **c**) and the Phe cluster near H_A_ that were substituted singly and in combination with Trp (**b**, **d**), in reference to the positions of the cofactors; **c** P^+^Q_B_^–^ yields in Phe-substituted RCs of the Trp cluster, studied in the YEFHV background (mean ± SD, *n* = 3 biologically independent experiments); **d** Rates and yields of initial ET and associated CR reactions in RCs carrying Trp substitutions of the Phe cluster residues, studied in the WT background with an active A path.

A striking break in the pseudo-C_2_ symmetry of the aromatic environment around the primary electron acceptors exists in RCs of all phototrophic bacteria of known sequence. In the structure of the *C. sphaeroides* RC, the environment of H_B_ features several tryptophans (Fig. 2a) whereas H_A_ is in proximity of multiple phenylalanine residues (Fig. 2b). These differences likely contribute to asymmetries in structure/function relationships on the two ET pathways. Mutants were constructed in which Trp residues at positions M127, M129, M130, and M148 near H_B_/Q_B_ (Fig. 2a) were replaced with Phe, both singly and in combination, in the YEFHV background. All but one of the single substitutions to Phe maintained or only slightly decreased the yield of B-side formation of P^+^Q_B_^–^ relative to that of the parent RC (YEFHV; Fig. 2c). Here, the PheM129 substitution had the largest negative effects. Any combination of the Trp cluster substitutions that contained the PheM129 substitution decreased the yield of P^+^Q_B_^–^ significantly and had large effects on the expression yields for RCs (Supplementary Table 2 and Supplementary Fig. 2).

In a similar—but opposite—approach, Phe residues L119, L121, and L123 near Q_A_ were substituted individually by Trp in the native RC background, and the pairwise and triplicate combinations were also constructed. The effects on the expression yields for RCs were small (Supplementary Table 3). The consequences of Phe → Trp substitutions on A-side ET demonstrate how subtle changes in aromaticity of the H_A_ binding pocket are reflected in the yield of P^+^H_A_^−^ (Supplementary Table 4 and Supplementary Fig. 3), the rate of ET from H_A_^−^ to Q_A_, and the rate of CR of P^+^Q_A_^−^, especially where the highly-conserved residue PheL121 was replaced with Trp (Fig. 2d). This substitution causes lengthening of the lifetime of P^+^Q_A_^–^ to ~140 ms vs. ~100 ms in the WT RC.

**Fig. 3:**
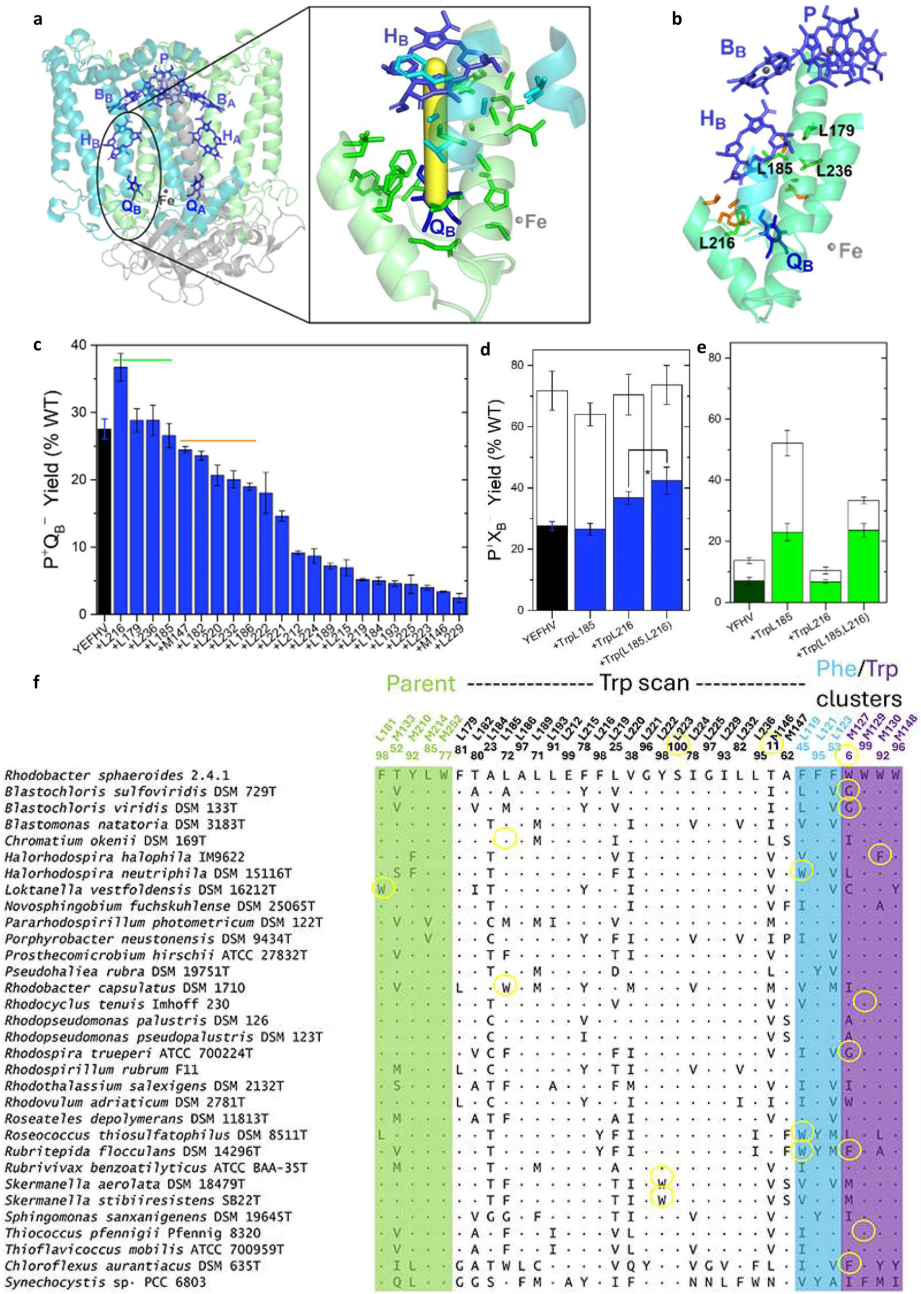
Consequences of alterations enhancing electron density between H_B_ and Q_B_ and conservation of amino acids at sites of substitution in native sequences of Type II reaction centers across phototrophic bacteria. A modeled “axis of interaction” (**a**, yellow) between H_B_ and Q_B_ relating residues on helices of the L (green) and M (cyan) subunits that were chosen for Trp-scanning mutagenesis. **b** Sites of the best-performing (green) Trp substitutions and other variants producing >70% of the P^+^Q_B_^–^ of the YEFHV parent (orange). **c** P^+^Q_B_^–^ yields of the Trp-scanning mutant RCs as measured by the millisecond assay (mean ± SD, n = 3 biologically independent experiments). Sites of Trp substitution in the YEFHV background are indicated, with sites identified in (**b**) encompassed by green and orange bars. **d**, **e** Yields of P^+^H_B_^–^ (open bars) and P^+^Q_B_^–^ (closed bars) in RCs carrying single and multiple Trp substitutions (**d**, YEFHV background; and **e**, YFHV background; mean ± SD, *n* = 3 biologically independent experiments), with significant differences from ANOVA indicated even when error bars overlap (* for *p* < 0.01). **f** Identities of amino acids at positions of substitutions examined in this study from a survey of 127 species of phototrophic proteobacteria (including analogous PS II in a cyanobacterium) where complete *pufL* and *pufM* sequences are available in genome databases^76^. The strains were selected as representatives of a subset of groups in recent phylogenetic classifications and/or key sequence variations that may have functional implications (yellow). Shown also are residues that comprise the sites of background mutations in the parent strains (green; Fig. 1d), sites associated with the Trp scan (black), as well as the Trp (purple) and Phe (cyan) clusters around the bacteriopheophytins. The overall conservation (%) of the *C. sphaeroides* amino acid at that site for this group of organisms is indicated at the top of the columns.

Conservation and differentiation of aromatic residues at these structural positions in the RC for all known sequences of phototrophic bacteria (Fig. 3; Supplementary Fig. 1) reveal that conservation of the Trp cluster is more strict than for the Phe cluster. The residue at position M127 is most variable, and curiously, a Gly residue can occur. A Phe residue can occupy all positions except M148. In contrast, the Phe cluster shows some plasticity in that smaller, aliphatic residues occur commonly at the L119 and L123 positions, with a natural frequency of TrpL119 that is nearly as high as that of GlyM127.

### Building a better tryptophan-assisted B-side ET pathway to Q_B_

A tryptophan-scanning mutagenesis strategy was employed to target the region of the RC complex between H_B_ and Q_B_ to discover a position(s) in the YEFHV parent RC where strategic placement of a tryptophan residue could enhance H_B_ → Q_B_ ET. Candidate sites for the introduction of Trp residues were identified by modeling a hypothetical axis of interaction between H_B_ and Q_B_ (Fig. 3a) using crystal structures of the native *C. sphaeroides* RC. Numerous factors are known to influence the amount of Q_B_ bound and its localization distal to the Fe, proximal to it, or distributed throughout the binding site^9,39–41^. Upon reduction via ET involving either A-side or B-side cofactors, the Q_B_ semiquinone anion occupies the proximal binding site, ~4.5 Å from the Fe atom^41,42^. Residues located within 6 Å of the modeled axis of interaction between H_B_ and Q_B_ bound in either the distal or proximal position were included in the initial list of target mutation sites. The candidate pool contained 33 residues (Supplementary Table 1) that clustered into distinct regions of the tertiary structure (Fig. 3a) and the primary sequence (Fig. 1e) of the RC. The majority lie on the transmembrane helices that form the interface between the L and M subunits in this region of the cofactor-binding interior of the RC complex.

Candidate residues in the parent YEFHV RC were substituted individually with tryptophan in order to determine the effect of the aromatic residue and its electron density between H_B_ and Q_B_ on yields of formation of P^+^Q_B_^–^ via B-side ET. Levels of RC expression in the mutant strains were variable, depending on the site of the Trp substitution (Supplementary Table 1; Supplementary Fig. 4a). Quantities of RCs sufficient for functional assays could be purified from 22 of the 33 mutant strains. When compared to the ground state spectrum of the parent YEFHV RC, the spectra of the 22 purified RCs (Supplementary Fig. 4c–f) show only slight shifts in the Q_y_ and Q_x_ transitions of the cofactors, indicating preservation of the integrity of the complex. For the other 11 candidates, expression levels were very poor, precluding purification of RCs for functional assays. The Trp substitutions in poor expressors tend to cluster at sites of subunit-subunit interaction in the hydrophobic helical interior of the complex or at sites of helix-cofactor (H_B_) interaction (Supplementary Fig. 4b).

Figure 3c shows the yields of P^+^Q_B_^–^ formed via B-side ET that were obtained for the mutants of the Trp-scanning set. The parent RC—YEFHV—is capable of using the B pathway to generate P^+^Q_B_^–^ at ~28% of the level achieved in the WT RC using the A-side cofactors. Of the 22 mutants screened, P^+^Q_B_^–^ is produced at equal or greater levels in RCs carrying a Trp substitution for either of four different residues—PheL179, LeuL185, PheL216 and LeuL236 (Fig. 3b). The amounts of P^+^Q_B_^–^ observed in the majority of the remaining Trp-scanning mutant RCs are well below that seen in the parent RC.

The TrpL216 substitution between H_B_ and Q_B_ is the most effective, increasing the yield of P^+^Q_B_^–^ to ~37%. Within the margin of error, Trp substitutions replacing PheL179, LeuL236, and LeuL185 slightly increased or preserved the ~28% level of B-side ET to Q_B_ that is seen in the YEFHV parent RC. These sites are quite distant from Q_B_ bound at the site that is proximal to the Fe atom (Fig. 3b). The side chain of LeuL185 is perpendicular to the center of the H_B_ macrocycle in the RC structure^31^. Modeling shows that particular rotamers of the side chain of the residue occupying the L185 site would be capable of interacting with the H_B_ macrocycle or one of its pyrrole rings^32^. PheL179 is positioned between B_B_ and H_B_ with its side chain pointing away from the cofactors towards the interior of the RC. LeuL236, on the E helix, is involved in weak interactions with the isoprenyl tail of Q_B_^43^.

Conservations of amino acids throughout phototrophic bacteria in the region of the RC complex (Fig. 3f) emphasize the flexibility of the tertiary structure of the B path. Trp residues occur naturally in some organisms at the L181, L185 and L222 sites. Surprisingly, ionizable residues, such as Asp at position L219, are also tolerated. A plethora of variability occurs in an adjacent hydrophilic region due to polarizable residues whose functions include binding and stabilization of Q_B_ and its intermediate redox and protonation states.

### Combined substitutions from the Trp-scanning set

Of the four Trp substitutions leading to the highest yields of P^+^Q_B_^–^, the expression levels of the YEFHV RCs that contain the TrpL216 or TrpL185 substitutions are reasonably high (Supplementary Table 5 and Supplementary Fig. 5a), facilitating their use in combination(s) with other promising substitutions. Thus, a strain was constructed in which the Trp substitutions at the L185 and L216 sites were combined in the YEFHV background and the effects on the yields of B-side ET to form both P^+^H_B_^–^ and P^+^Q_B_^–^ are shown in Fig. 3d. The combination of the two Trp substitutions—one near H_B_ and one between H_B_ and Q_B_—does not change the amount of P^+^H_B_^–^ significantly from that observed for the parent YEFHV RC ( ~ 72-74%^32,33^). However, addition of the two Trp substitutions to the YEFHV background results in a RC where the overall yield of B-side formation of P^+^Q_B_^–^ is increased to a level that is 42% of the amount that is formed via A-pathway ET in the WT RC, representing a ~ 1.5-fold increase over the amount ( ~ 28%) formed by the YEFHV parent RC. An overall ~42% yield of P^+^Q_B_^–^—when combined with the ~72–74% yield of P^+^H_B_^–^ in that RC^31^—translates to a ~ 57% yield of secondary ET from P^+^H_B_^–^ to P^+^Q_B_^–^, as compared to the ~40% yield of secondary ET seen in the YEFHV parent RC and in other RCs that have native residues surrounding H_B_ and Q_B_^31,32^.

The effects of single or combined TrpL185 and TrpL216 substitutions were examined in a second type of parent RC that did not carry the GluM133 background substitution (the “E” mutation in the YEFHV RC; Fig. 3e). The ‘YFHV’ parent RC of this set (Fig. 1d) is characterized by a small ~14% yield of P^+^H_B_^–^ that results in 7% overall yield of P^+^Q_B_^–^ formed by B-side ET^28^ as compared to ~28% overall yield of P^+^Q_B_^–^ for the YEFHV parent (Fig. 3d). As shown in Fig. 3e, addition of the TrpL216 substitution to the YFHV parent RC had little to no effect on P^+^Q_B_^–^ yield. But addition of the TrpL185 substitution had a dramatic effect in the YFHV background, increasing the yield of P^+^Q_B_^–^ by ~3.5-fold to a level that is ~25% of the amount of P^+^Q_B_^–^ formed by A-branch ET in native RCs. When present in the YFHV background (Fig. 3e), the TrpL185 substitution increases the amount of P^+^H_B_^–^ that results from B-side ET to ~52% over a yield of ~14% that forms in its absence^33^; secondary ET then converts about half of P^+^H_B_^–^ to P^+^Q_B_^–^. Combination of Trp substitutions at both the L185 and L216 sites with the YFHV background mutations did not result in any further increase in the amount of P^+^Q_B_^–^ formed and may have had a slight negative effect (within error; Fig. 3e).

In contrast, addition of the TrpL185 substitution to a GluM133-containing RC (the YEFHV parent) had little to no impact on the yield of P^+^Q_B_^–^ (Fig. 3d), a result that is contrary to its effects described just above in the YFHV background (Fig. 3e). Amino acid side chains at both the L185 and M133 sites approach the tetrapyrrole rings of H_B_, but from different orientations. As evidenced by a red shift of ~8 nm in the Q_x_ absorption band of H_B_ in ground state spectra, the side chain of GluM133 likely forms a hydrogen bond with the keto substituent of ring V^28,44,45^. This interaction stabilizes P^+^H_B_^–^, increasing its yield and the overall yield of P^+^Q_B_^–^^28,44,45^. In the context of the YEFHV RC, P^+^H_B_^–^ is so well stabilized that any potential effect of the TrpL185 substitution is masked by the influence of the GluM133 substitution^32,33^.

### Secondary ET to Q_B_ via redesigned pathways

The observations that the TrpL216 substitution (in tandem with the YEFHV background mutations) does not negatively impact the structural integrity or occupancy of the Q_B_ site of the *C. sphaeroides* RC are corroborated by additional independent evidence obtained from a photocompetent phenotypic revertant strain. A multiply-passaged culture of the photosynthetically-incompetent strain carrying the TrpL216 substitution in the YEFHV background (Fig. 3c, d) was subjected to selective photosynthetic growth conditions on agar plates, and a few photocompetent colonies appeared. Sequence analysis of plasmid DNA isolated from these phenotypic revertant colonies identified a single mutation that replaced ValM252 with Phe, negating the engineered blockage of Q_A_ binding (Fig. 1d) and re-opening the A-side ET path for reduction of Q_B_. These results align with previous work^46,47^ showing that aromatic residues Phe and Tyr support photosynthetic growth when used to replace the native TrpM252 (M250 in *R. capsulatus*). Indeed, results show that the ‘normal’ A-path photocycle, culminating in ET from Q_A_ to Q_B_, is restored in the RC purified from the revertant strain that grows photosynthetically, confirming that Q_B_ can function effectively as a 2e^–^/2H^+^ acceptor within an altered binding pocket that carries the TrpL216 substitution.

**Fig. 4:**
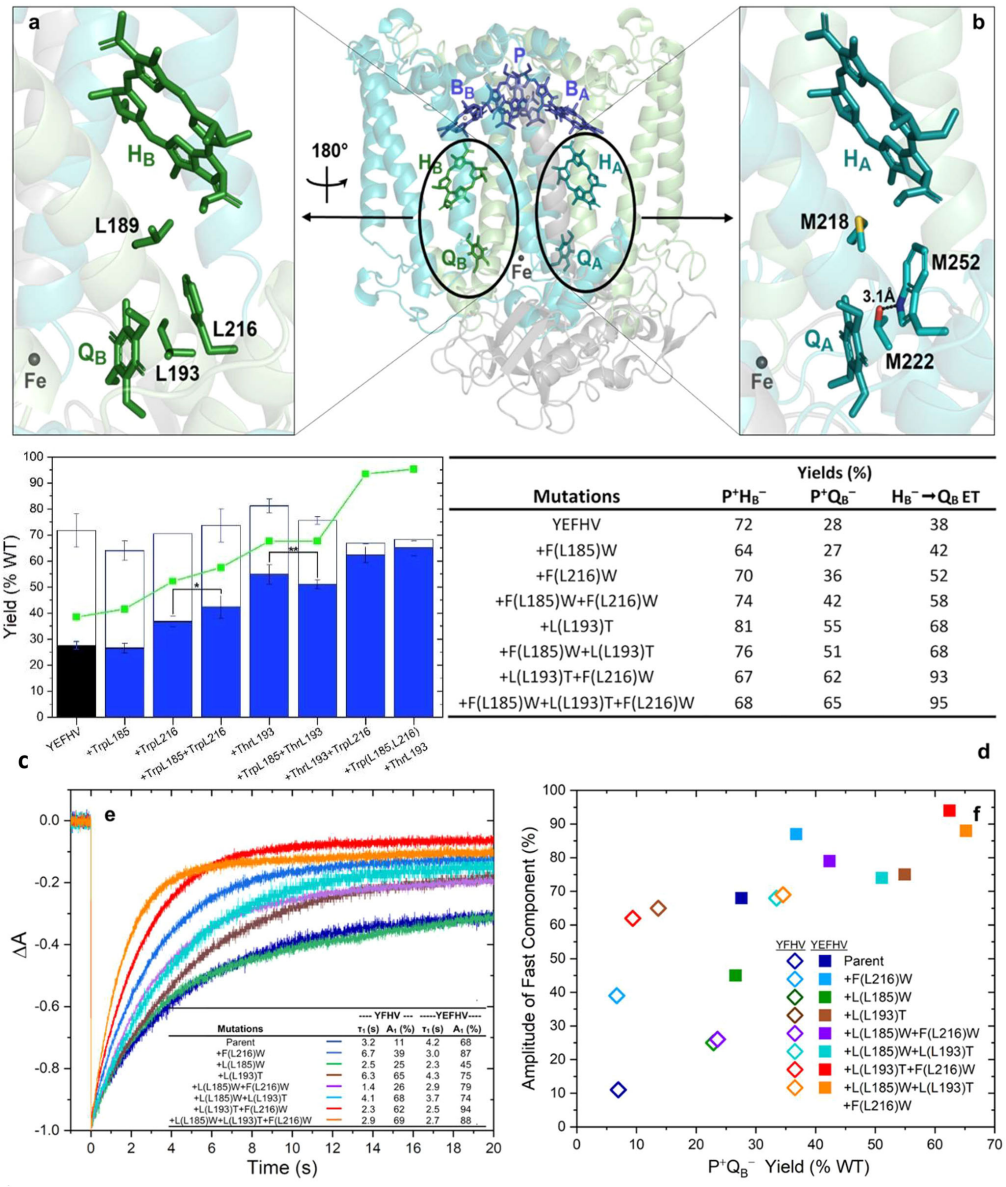
Optimization of secondary B-side ET through introduction of a nearby Threonine substitution and P^+^Q_B_^–^ charge recombination kinetics, reflecting increased electronic coupling of B-side cofactors. PheL216 and LeuL193 residues near Q_B_ (**a**, with the complex rotated 180 degrees), highlighting C_2_-symmetry-related similarities and differences between the quinone-binding pockets on the A and B paths. **b** The conserved amino-acids (ThrM222, TrpM252) and cofactor interactions near Q_A_ that affect ET from H_A_. **c** Yields of P^+^H_B_^–^ (open bars) and P^+^Q_B_^–^ (closed bars) formed by B-branch ET in RCs carrying mutation(s) from the Trp-scanning set—with or without the ThrL193 substitution (mean ± SD, *n* = 3 biologically independent experiments) – as well as the yield of the secondary H_B_^─^ → Q_B_ ET (green). Significant differences from ANOVA indicated when error bars overlap, * for *p* < 0.01 and ** for *p* < 0.03. **d** ET yields in B-side enabled mutant RCs. **e** Charge recombination traces (for RCs constructed in the YEFHV background) showing the complex nature of all kinetics (best described by multiple exponentials) to indicate that, at sufficiently long times, all curves converge to ΔA = 0. Inset: Time constants and amplitudes of the fast component of P^+^Q_B_^–^ charge recombination from RCs prepared from cultures harvested in mid- to late-log phase (Supplementary Fig. 7). **f** The relationship between the amount of P^+^Q_B_^–^ formed via B-side ET and the fast component of CR of that state in selected mutant RCs.

Further remodeling of the B-side secondary ET pathway in a TrpL216-containing YEFHV RC was inspired by inspection of the Q_A_ and Q_B_ binding pockets in crystal structures of the native *C. sphaeroides* and related *Blastochloris* (*B*.) *viridis* RCs where notable differences between interactions of each quinone with its neighboring amino acids are apparent (Fig. 4a, b). Near Q_A_, strictly-conserved TrpM252 participates in π electron-mediated interactions with both Q_A_ and H_A_^9,47,48^. ThrM222 (Fig. 4b)—also strictly conserved—forms a hydrogen bond with the indole nitrogen of TrpM252, positioning the large aromatic side chain in an offset parallel interaction for substantial orbital overlap with the quinone ring. This interaction enhances Q_A_ binding and promotes rapid ET to it from H_A_^−^^47^. In the Q_B_ site of the native RC, highly-conserved residue LeuL193—symmetry-related to ThrM222 in the Q_A_ site—is positioned between H_B_ and Q_B_ where it has potential to interact with PheL216 (Fig. 4a). However, no hydrogen-bonding interaction of a type similar to that seen in the native RC for TrpM252-ThrM222 near Q_A_ (Fig. 4b) can occur between the side chains of PheL216 and LeuL193 in the Q_B_ site (Fig. 4a).

Accordingly, the ThrL193 substitution was added to the (i) the parent YEFHV RC, and combined (in the YEFHV background) with Trp substitutions at (ii) L185, (iii) L216, and (iv) both sites. Figure 4c shows the results of these substitutions on the yields of B-side formation of both P^+^H_B_^–^ and P^+^Q_B_^–^. As expected, when compared to the YEFHV parent, the substitutions above did not increase the ~65–75% yield of P^+^H_B_^–^ significantly (Fig. 4c, open bars). In contrast, the effects of the added substitutions on the yield of P^+^Q_B_^–^ are dramatic (Fig. 4c, solid bars) with corresponding increases in secondary ET from H_B_^–^ to Q_B_ (Fig. 4c, green line). The addition of ThrL193 on its own (or in combination with the TrpL185 substitution) to the YEFHV RC increased the yield of B-side formation of P^+^Q_B_^–^ by almost twofold to ~55% (from 28%) of the amount formed from A-side ET in the native RC. Remarkably, the amount of P^+^Q_B_^−^ is boosted ~2.2-fold to ~62–65% in YEFHV RCs that carry the ThrL193-TrpL216 combination. The positive effect of the ThrL193-TrpL216 combination is noted for YEFHV RCs that carry either the native Leu or a Trp substitution at L185 (Fig. 4c, two right bars). Substitution of the smaller serine residue at L193 has a lesser effect, if any (Supplementary Table 5 and Supplementary Fig. 5a–d).

The pairing of ThrL193-TrpL216 – with or without TrpL185—leads to a striking increase in the rate of secondary ET such that it outcompetes CR of P^+^H_B_^–^ to the ground state. The decay of P^+^H_B_^–^—via ET to form P^+^Q_B_^–^ or CR to the ground state—for each mutant RC is shown as the branching yield for H_B_^–^ → Q_B_ in Fig. 4c and summarized in Fig. 4d. Within the series of mutant RCs in Fig. 4c, the yield of the secondary H_B_^–^ → Q_B_ ET reaction more than doubles from ~40% in the YEFHV parent to ~95% in the two RCs carrying the ThrL193-TrpL216 combination of substitutions (Fig. 4c, green line).

### Rates of charge recombination of the P^+^Q_B_^–^ state—relationship to P^+^Q_B_^–^ yield

The Trp and Thr substitutions also affect the kinetics of the P^+^Q_B_^–^ charge recombination reaction. As shown in Fig. 4e, f, the P^+^Q_B_^–^ state formed via B-side ET is long-lived, unlike the P^+^Q_A_^–^ state that results from ET via a native A-side pathway ( ~ 100 ms lifetime, if Q_B_ is absent; Fig. 2d). The kinetics of P^+^Q_B_^–^ CR are complex^16,49^ and cannot be described by a single exponential. RC samples displaying the most uniform kinetics were purified from cultures harvested in mid- to late-log phase (Supplementary Fig. 7). The kinetics are characterized by a fast component on the timescale of a few seconds (Fig. 4e) and a slow component(s) that can range from ~20 s to greater than 100 s. Fast components were observed to dominate CR kinetics as the substitutions increased the yield of the secondary B-path ET reaction, with kinetics for all of the mutant RCs being faster than that of the YEFHV parent RC (Fig. 4e).

Figure 4f shows the relationship between the fraction of P^+^Q_B_^–^ that recombines on the fast timescale of a few seconds and the yield of the state produced by forward ET. At the lower extreme of both axes is the original precursor YFHV RC^28^. Because the RC carries only the swap of the PheL181-TyrM210 conserved asymmetry (Fig. 1d), a low level of B-side formation of P^+^Q_B_^–^ (7%) was observed^28^. The decay of that state occurs with very slow overall CR kinetics that are characterized by negligible amounts of a fast component. The addition of one or more substitutions that stabilize P^+^H_B_^–^—GluM133^28^ and/or TrpL185^32,33^ (Fig. 3c)—boosts the amount of the primary B-side ET processes P* → → P^+^H_B_^–^ but produces only a proportional rise in the yield of P^+^Q_B_^–^. It is notable that both the GluM133 and/or TrpL185 substitutions are linked to a corresponding increase in the amplitude of the fast component of the superexchange-mediated P^+^Q_B_^–^ CR, perhaps involving P^+^H_B_^–^ as a virtual intermediate. The effect of the GluM133 substitution is substantial, while the influence of the L185W substitution is readily apparent in the YFHV background (in the absence of GluM133).

Increases in both the yield of P^+^Q_B_^–^ and the CR rate are afforded by joining TrpL216 to previous substitutions that enable substantial B-side ET to H_B_. Mutant RCs that are characterized by the most rapid overall P^+^Q_B_^–^ CR kinetics are those which merge the ThrL193 substitution with one or more Trp substitutions on the ET pathway—ThrL193-TrpL216+YEFHV and TrpL185-ThrL193-TrpL216+ YEFHV—to produce the greatest overall yield of P^+^Q_B_^–^ (60-65%) (Fig. 4c, d). Fast components dominate the CR kinetics overwhelmingly ( > 80%) in these RCs where yield of the H_B_^–^ → Q_B_ secondary ET step is now ~95%.

## Discussion

After decades of spectroscopic investigations of bacterial photosynthetic RCs that demonstrated activity of a single transmembrane ET pathway, the subsequent realization of the RC structure^8^ revealing two identical sets of cofactors branching from a central Bchl dimer was an enigma. Dual sets of cofactors are now known to be common to all RCs. While both branches of cofactors operate in type I RCs, type II RCs use only the A-side cofactors for the initial transmembrane charge separation reactions (reviewed in Ref. ^11^). The optimization of the A pathway and the deactivation of the B pathway from an ancestral dual-pathway RC occurred over three billion years. Thus, (re)vitalization of the inactive B pathway to produce a high yield of transmembrane charge separation entails more than substitution of one or a few residues. It requires multi-site manipulation of protein-cofactor interactions to rebalance energy levels of charge-separated states to both disable the A pathway and overcome existing B-path barriers to achieve a stable B-side transmembrane charge separation.

Previously, several iterations of mutant RCs were constructed and characterized to identify variants in which A-side primary ET was largely impaired, secondary A-side ET to Q_B_ was blocked, and the early steps in B-path charge separation were activated^27–30^. Additional mutagenesis increased the yield of the initial stable charge-separated state, P^+^H_B_^–^, to 70-90% of the amount of P^+^H_A_^–^ that is formed by A-side ET in the native RC^31–34^. However, the yield of the subsequent H_B_^−^ → Q_B_ secondary ET reaction is only ~40% in RCs with native residues surrounding H_B_ and Q_B_. This shortcoming is especially perplexing in that the the driving force/free energy for secondary ET on the B path is significantly larger (by 210 meV; Fig. 1b) than the same on the A side (with P^+^H_B_^–^ known to be at higher energy than P^+^H_A_^–^ with P^+^Q_B_^–^ at lower energy than P^+^Q_A_^–^). Consequently, P^+^H_B_^−^ → P^+^Q_B_^−^ secondary ET reaction was targeted for improvement in this study as a necessary step in achieving a RC that uses the B pathway solely for light-driven, transmembrane charge separation.

To realize a high yield of secondary ET on the B path, additional engineering must address the separation of H_B_ and Q_B_ by ~10 Å, forbidding the potential for direct orbital overlap. Aromatic residues – especially phenylalanine, tyrosine, and tryptophan – often assume a role to bridge large distances between ET cofactors, serving in some cases as redox-active intermediates^1–3^ or as mediators of superexchange^4–6^. The frontier molecular orbitals of aromatic residues are closer in energy to those of the conjugated cofactors, allowing stronger interactions than are possible with the side chains of aliphatic amino acids. Conspicuous clusters of Phe residues between H_A_ and Q_A_ on the optimized A-side pathway (Fig. 2b) and Trp residues between H_B_ and Q_B_ on the inactive B-side pathway (Fig. 2a) were targeted with Trp or Phe substitutions, respectively. The sequence comparisons (Fig. 3f), combined with the consequences of the these substitutions on yields of P^+^H_A_^−^ states and yields of A-side secondary ET (Fig. 2d), suggest a role for the native Phe residues in the *C. sphaeroides* RC in promoting rapid, complete ET through the A-path cofactors. Functional characterization of the mutant RCs also suggests that the larger dielectric presented by the B-side Trp cluster function in the native RC may stabilize P^+^Q_B_^−^ as an intermediate in the two-electron photocycle. It is noteworthy that substitutions of Trp-cluster residues—some of which are highly conserved (Fig. 3f)—demonstrated large negative effects on RC expression yields (Supplementary Table 1; Supplementary Fig. 1b). This underpins their role in providing structural stability to that region of the complex, in contrast to any structural role of the Phe residues surrounding the primary acceptor on the A side (Supplementary Fig. 2b).

The Trp-scanning approach was effective in discovering a position(s) in the YEFHV parent RC where strategic placement of a tryptophan residue could alter the interplay between ET and CR to favor conversion of state P^+^H_B_^−^ (Fig. 1b) by forward ET to produce more P^+^Q_B_^−^. Four different sites were identified where a Trp substitution resulted in a RC that produced a yield of P^+^Q_B_^−^ from a single excitation of P that is equivalent to or greater than that of the parent RC. The mechanisms by which the accumulation of P^+^Q_B_^−^ is affected are likely to be diverse because a Trp substitution can influence multiple properties, including the positioning and redox potentials of cofactors, coupling between them, and/or Q_B_ binding.

The site of the most effective Trp substitution is at position L216, where the native highly-conserved Phe residue is located between H_B_ and Q_B_ (Fig. 4a). The aromatic ring of PheL216 (symmetry-related to TrpM252) is within van der Waals bonding distance of and is involved in an offset parallel, π-electron-mediated interaction with the ring of Q_B_ (Fig. 4a)^9,41^ that is critical for its binding^50^. Stronger interactions of this type occur between PheL216 and aromatic triazine herbicides that displace Q_B_; in RCs of *B. viridis*, herbicide resistance results from elimination of the ring-ring interaction with the triazine molecule by mutation of PheL216 to Ser^50–52^. As shown above, substitution of a Trp for PheL216 in the YEFHV background leads to an increase in the yield of B-side formation of P^+^Q_B_^−^ on its own or when paired with a Trp substitution at a second site (L185) near H_B_ (Fig. 3c,d). When constructed in the YEFHV background, neither of these Trp substitutions increases the amount of P^+^H_B_^–^ (Fig. 4d), but they do affect the decay of P^+^H_B_^–^ to favor ET to Q_B_ over CR to the ground state.

Substitutions designed to assist Q_B_ in functioning effectively as a mobile, two-electron acceptor in a revitalized B-side ET pathway can influence many aspects of quinone binding and photochemical properties. A redesigned Q_B_ site must strike a balance between enabling oxidized Q_B_ to enter the RC and move to the proper position to accept an electron from H_B_^−^, stabilizing its semiquinone intermediate species, and ensuring that its fully-reduced/fully-protonated form can leave the RC to complete the cyclic-ET process and be replaced by a oxidized molecule from the membrane pool. Modifications of residues in a binding pocket can have dramatic effects on the quinone’s redox potential, interactions important for binding of both neutral and semiquinone forms, pK_a_s of the quinone forms and nearby residues, and the network of water molecules and polar side chains that comprise the pathways for proton donation to the Q_B_ anions in any modified RC that would be capable of cyclic B-side ET. In the Q_A_ site, Stilz et al.^47^ showed the importance of ThrM222 (Fig. 4b) for quinone binding as its replacement by Val caused partial loss of Q_A_ without altering the rate of P^+^H_A_^–^ → P^+^Q_A_^–^ ET in the *C. sphaeroides* RC. Thus, its symmetry-related counterpart in the Q_B_ site—LeuL193 (Fig. 4a)—was replaced by Thr. As described above, the ThrL193 substitution in the YEFHV RC leads to a substantial increase in the yield of P^+^Q_B_^−^ (Fig. 4c). The proximity of ThrL193 to Q_B_ (Fig. 4a) affords the possibility for formation of a hydrogen bond between the –OH group of the Thr and a keto group of the quinone. As compared to the native Leu, both the polarity and reduced volume of the Thr side chain could lead to alterations of any or all of the above list of interactions in the Q_B_ site that determine quinone binding and redox chemistry. In the ThrL193+YEFHV mutant RC, the ThrL193-(native)PheL216 pairing in the Q_B_ site is analogous to the Q_A_ site in other mutant RCs of both *C. sphaeroides*^47^ and *R. capsulatus*^53^ that combined the native ThrM222 (M220 in *R. capsulatus*) with the Phe substitution of the native TrpM252 (M250). These latter mutant RCs are characterized by reduced Q_A_ binding and a H_A_^–^ → Q_A_ ET rate that is ~4-6-fold slower than native, but they could still support photosynthetic growth of either organism^47,53^ (as also seen in the phenotypic revertant strain described above).

The largest yield of B-side formation of P^+^Q_B_^−^ in these sets of mutant RCs is achieved when the ThrL193 substitution is coupled in the YEFHV background with either the TrpL216 or TrpL185-TrpL216 substitutions. It is unlikely that TrpL185 and ThrL193 sites would interact significantly due to the distance between them ( > 10 Å). But, when ThrL193 is present in combination with TrpL216, the capability exists for formation of a hydrogen bond between the threonine –OH group and the indole nitrogen of the tryptophan. This hydrogen bond likely reduces the degrees of freedom of the TrpL216 side chain and strengthens its π-orbital-mediated, ring-ring interactions with Q_B_, serving to ‘tether’ Q_B_ in its binding site in an orientation that promotes ET from H_B_. The plasticity of this region of the protein and the observation that more than one mutant RC complex is capable of high yields of secondary B-path ET (Fig. 4c,d) allows for flexibility in future engineering as other functional properties of Q_B_ and its redox intermediate states are balanced and optimized cooperatively. Interestingly, this structural and functional malleability does not appear to be readily apparent on the A path (where changes would be detrimental rather than beneficial).

From a simplistic standpoint, the amino acid substitutions present on the B side of the RC in the variants with the highest yields of P^+^Q_B_^−^ mirror, in part, some features of the native A-side ET pathway. The most recent substitutions that were introduced have greatly increased the yield of the secondary H_B_^−^ → Q_B_ ET step by duplicating in the Q_B_ site some important structural interactions from the Q_A_ binding site (Fig. 4b). But the TrpL185 substitution is a notable exception to this ‘duplication’ concept. TrpL185 serves to stabilize P^+^H_B_^−^ in the *C. sphaeroides* RC^32,34,54^, but Trp is never found in the symmetry-related M214 posotion near H_A_ on the A-side ET pathway (Fig. 3f; Supplementary Fig. 1). Trp is found naturally at the L185 position in *R. capsulatus* and several members of the Chloroflexota. Another unexpected result was the identification of TrpL216 as a beneficial substitution in the Trp-scanning experiment in light of its deleterious effects in the *R. capsulatus* RC where it was an early target for mutagenesis strategies to increase the yield of P^+^Q_B_^–^ from B-side ET^29^. However, in that organism, a TrpL216 substitution was not pursued further after results showed that it significantly reduced B-side formation of P^+^Q_B_^–^ in RCs carrying two different sets of background mutations, suggesting adverse effects on Q_B_ binding.

In the native RC, P^+^Q_B_^−^ is formed from P^+^Q_A_^−^, and there are two routes for CR of P^+^Q_B_^−^. The proportion P^+^Q_B_^−^ of that decays via one route or the other is controlled by factors that influence the free energy gap between the quinones^16,55^. Under physiological conditions in native RCs, a small fraction of P^+^Q_B_^−^ recombines via a “direct”, B-side superexchange route involving orbitals of H_B_, B_B_ and the intervening amino acids as virtual mediators. The majority of P^+^Q_B_^−^ recombines by an “indirect” pathway in which P^+^Q_A_^−^ is thermally repopulated and then decays subsequently by a direct A-side superexchange route involving H_A_, B_A_, and neighboring amino acids.

In the mutant RCs of this study, P^+^Q_B_^−^ generated by B-side ET decays solely via a direct B-side route through the protein without repopulation of the intermediate P^+^H_B_^−^ or P^+^B_B_^−^ states (which are thermally inaccessible). This B-side CR route for P^+^Q_B_^−^ is analogous to the A-side route for the direct decay of P^+^Q_A_^−^. Despite the identity of donor/acceptor cofactors and twofold symmetry in orientations and spacings on both the routes (Fig. 1a), the rates of direct CR under physiological conditions for the P^+^Q^–^ states on either side of the native RC differ by more than one order of magnitude [P^+^Q_B_^–^, ~(8 s)^-1^; P^+^Q_A_^–^, ~(120 ms)^-1^;Fig. 1b;^16,55^]. The large difference in the direct CR rates in native RCs has been attributed to both greater mixing between the charge-separated states on the A side and greater reorganization energy on the B side that is associated with the abundance of polar and polarizable residues and water molecules in the Q_B_ binding site^9^ (and others). The effective electronic mixing between charge-separated P^+^Q^–^ and ground PQ states for a CR process (on either the A or B branches) can be described by perturbation theory with an expression that depends on two factors^56,57^. The numerator reflects the effective electronic interactions that reflect spatial orbital overlaps involving the cofactors (P, B, H, Q) and the properties of the amino acids in the intervening medium − the larger the electronic interactions, the faster the rate. The denominator involves the free energies of the various virtual intermediates that mediate the superexchange process (e.g., P^+^B^−^ and P^+^H^−^), which can be modulated by the amino acid substitutions incorporated − the smaller the energy denominator, the faster the rate. CR kinetics for P^+^Q_B_^−^ generated by B-path ET were examined in several mutant RCs of this study that differ in the yield of the secondary H_B_^–^ → Q_B_ ET step (Fig. 4d). The progressive enhancements in this ET reaction correlate with increases in the overall rate of P^+^Q_B_^–^ CR because the same factors affect both superexchange-mediated processes.

In particular, the TrpL185/L216 and ThrL193 substitutions in this study that dramatically increase the yield of P^+^Q_B_^–^ likely produced that result not only by increasing Q_B_ binding but by improving electronic coupling between Q_B_ and H_B_, and perhaps altering the free energies of P^+^H_B_^–^ and P^+^Q_B_^–^. Although the kinetics of CR of P^+^Q_B_^–^ are multiphasic, the fraction of the component with the shortest lifetime grows in proportion with increases in the conversion of P^+^H_B_^–^ to favor ET to Q_B_. The increases in the rate of CR are likely a result of enhanced orbital overlaps mediated by substitutions in the inter-cofactor space between the B-side cofactors. The effects of slight polarity changes in the substitutions on reorganization energy are not expected to be significant. The nonlinear empirical relationship between yield of P^+^Q_B_^–^ and the amplitude of fast CR kinetics (Fig. 4e, f) may stem, however, from large reorganizations of interconnected networks of polar side chains and water molecules in the Q_B_ binding pocket following the generation of the semiquinone anion. These events may mimic those observed in other situations that increase the free energy gap between Q_B_ and Q_A_ – e.g., WT RCs at high pH^58^, WT RCs with low potential quinones substituted in the Q_A_ site^55^, or mutant RCs^59–61^ – such that P^+^Q_B_^–^ (formed in these cases from P^+^Q_A_^–^) recombines directly through the B-side protein/cofactor milieu due to an inability to repopulate P^+^Q_A_^–^.

## Outlook

In cells expressing the mutant RCs that perform B-side transmembrane charge separation with 65% overall efficiency, the lifetime of P^+^Q_B_^–^ ( ≥ ~1.5 s; Fig. 4e) is more than sufficient for the semiquinone anion to become stabilized by initial proton uptake and outlast the process of reduction of P^+^ to ground-state P by periplasmic cytochrome *c*_2_ occurring on the microsecond timescale^62^. These characteristics would ready the stabilized semiquinone intermediate for acquisition of another electron via B-side ET following a second excitation of P as a necessary step in 2e^–^/2H^+^ photochemistry. These mutant RCs are promising stepping stones towards the realization of subsequent *C. sphaeroides* variants in which cyclic B-side ET supports photosynthetic growth of the organism. In addition, with advancements in application of strain engineering approaches to newly discovered organisms (or those potentially unculturable in the laboratory and/or less amenable to genetic manipulation), opportunities exist to test the universality of these results in the RC machinery of other species.

## Methods

### Construction, Expression and Purification of Mutant Reaction Centers

*C. sphaeroides* mutant RCs were constructed using a cassette-based mutagenesis platform based on a derivative of the engineered plasmid pBBRRWsLsM carrying genes of the *puf* operon^28^. For mutagenesis of “Phe cluster” residues, a silent *Bst*EII site was introduced within the codon for L114Gly via site-directed mutagenesis (QuikChange) of pBBRRWsLsM (Fig. 1e). Subsequently, synthetic oligonucleotide cassettes (Eurofins) were designed to contain the desired Phe cluster mutations singly and in combination between *Bst*EII/*Nru*I sites. For the “Trp cluster” mutant set, the *Bmt*I*-Avr*II segment (69 bp; M127, M129, M130) of pBBRRWsLsM derivatives was replaced with two overlapping cassettes to substitute Phe at these sites individually and in combination; residue M148 lies within overlapping cassettes flanked by *Avr*II and *Stu*I sites. “Tryptophan scanning” mutants (Supplementary Table 1) comprised 33 residues clustered into four distinct regions within the RC primary sequence and tertiary structure. Strategies for cloning of synthetic mutant cassettes were specific to each region of the primary sequence (Fig. 1e): Region DI, *Hin*dIII-*Bbv*CI cassette; Region JEK, two overlapping cassettes that spanned the *Bsi*WI-*Afl*II segment (86 bp); Region GA (75 bp), two overlapping cassettes flanked by *Avr*II and *Stu*I sites; Region H, *Age*I-*Hpa*I cassette.

Synthetic cassettes were ligated to derivatives of plasmid pBBRRWsLsM2 carrying the desired background substitutions (Fig. 1d). Candidate mutant plasmids isolated from *E. coli* DH5α transformants were identified with restriction enzyme screening. Sequence-verified plasmids were then transferred to *C. sphaeroides* expression host ΔrshI via electroporation^28,63^. Estimates of RC expression yields were obtained from small-scale (80 ml) chemoheterotropic cultures (Supplementary Fig. 2). All cultures for RC purification were grown chemoheterotrophically in 2 L of medium (in a 2.8 L baffled Fernbach flask with a silicone sponge closure, 125 rpm, 33 C, in the dark^28^). His-tagged mutant RCs were solubilized from the antennaless, ‘RC-only’ membranes of the ΔrshI host strain with the mild detergent Deriphat 160-C that is known to preserve occupancy of the Q_B_ site^49^. RCs were purified with immobilized metal affinity chromatography and concentrated in a manner consistent with previous practices^32,54^ for use in biophysical assays to characterize their photochemistry. *E. coli* strains were grown on LB medium. *C. sphaeroides* strains were cultured on ^G^YCC medium (Sistrom’s YCC^64^ containing an additional 1 g/L of yeast extract and with the pH adjusted to 7.1). The plasmid was selected by growth on media containing gentamicin (12 µg/mL for *E. coli*, 24 µg/mL for *C. sphaeroides*).

None of the mutant strains constructed in this study are capable of photosynthetic growth. For the selection of photocompetent phenotypic revertants of the F(L216)W + YEFHV mutant strain (S103; Supplementary Table 1), a culture was passaged through several rounds of growth in the dark (in the absence of selection for photosynthetic capability), serial dilution, and regrowth. An aliquot was then plated onto ^G^YCC agar and the plate was transferred to photosynthetic growth conditions (anaerobic chamber, 850 nm illumination, 33 C). After several days of incubation, photocompetent colonies were observed. The plasmid carrying the *pufLM* genes was recovered from those colonies and subjected to DNA sequence analysis (University of Chicago Comprehensive Cancer Center Facility).

### Functional Characterization of Mutant RCs

Ground state spectra of purified RCs and cleared lysates of cell cultures were obtained at room temperature^28^. The relative yields of Q_B_ reduction via B-side ET in mutant RCs lacking Q_A_ were assessed rapidly with a plate-based, time-resolved screening assay (millisecond resolution) to measure photobleaching of P^29^. Since Q_A_ is absent due to the ValM252 substitution, the signal on this timescale indicates formation of P^+^Q_B_^−^ solely via the activity of the B-side pathway. The yield of P^+^Q_B_^−^ is reflected by the amplitude of P-bleaching measured at 850 nm, 1 ms following a 7-ns 532-nm excitation flash. The kinetics of P^+^Q_B_^–^ charge recombination were monitored using the same setup.

To assess the yields of P^+^H^−^ species formed via A- and B-side primary ET, selected WT and mutant RCs were subjected to ultrafast transient absorption experiments conducted using 100 femtosecond pulses from a 850-nm laser system (Spectra Physics) in conjunction with Helios or EOS spectrometers and standardized approaches for data analyses^28,54^. The yields of P^+^H^−^ charge-separated states were obtained by integrating the maximal bleaching (acquired at 250 ps) of the Q_x_(0,0) and (1,0) vibronic manifold, harmonized with approaches for other mutant RCs^31,33,34^. Kinetic data were evaluated at individual wavelengths and/or by global analysis^33^.

### Reporting summary

Further information on research design is available in the Nature Portfolio Reporting Summary linked to this article.

## Supplementary information

Transparent Peer Review file

Supplementary Information

Reporting Summary

## Data Availability

All unique and stable reagents will be made publicly available, some will require Material Transfer Agreements. Underlying source data are available with the manuscript online.
