# Supplementary material for: Inter-cofactor protein remodeling rewires short-circuited transmembrane electron transfer
Source: Commun Chem. 2025 Apr 9;8:110. doi: 10.1038/s42004-025-01460-y (PMC11982316; doi:10.1038/s42004-025-01460-y)

Supplementary Information

Inter-cofactor protein remodeling rewires short-circuited transmembrane electron transfer

Deborah K. Hanson[1,2], James C. Buhrmaster[1,2], Ryan M. Wyllie[1,2], Gregory A. Tira[1], Kaitlyn M. Faries[3], Dewey Holten[3], Christine Kirmaier[3], Philip D. Laible[1,*]

Correspondence to: laible@anl.gov

[1]Biosciences Division, Argonne National Laboratory, Lemont, IL 60439. [2]These authors contributed equally: Deborah K. Hanson, James C. Buhrmaster, Ryan M. Wyllie. [3]Department of Chemistry, Washington University, St. Louis, MO 63130. *email: laible@anl.gov

Supplementary Figure 1 | Identities of amino acids at positions of substitutions.
Survey of 127 species of phototrophic proteobacteria (including analogous systems in PS II found in eukaryotes) where complete *pufL* and *pufM* sequences are available in genome databases. Highly conserved residues that comprise the sites of background mutations in the parent strains (green, sites associated with the Trp scan (black), as well as the Trp (purple) and Phe (cyan) clusters around the bacteriopheophytins are shown with the overall conservation (%) of the *C. sphaeroides* amino acid at that site for this group of organisms is indicated at the top of the columns.

Parent --------------- Trp scan ------------- Phe/Trp clusters

Species	Residues
Position	L181 M133 M210 M214 M252 L179 L182 L184 L185 L186 L189 L193 L212 L215 L216 L219 L220 L221 L222 L223 L224 L225 L229 L232 L236 M146 M147 L119 L121 L123 M127 M129 M130 M148
Conservation (%)	98 53 95 84 77 82 80 23 73 98 73 91 99 76 98 26 39 98 98 100 79 99 95 81 96 11 62 45 94 53 6 98 95 98
Cereibacter sphaeroides 2.4.1	F T Y L W F T A L A L L E F F L V G Y S I G I L L T A F F F W W W W
Acidiphilium acidophilum DSM 700	· I · · · L · T F · · · · · · F I · · · · · · · · · · A · V I · · ·
Acidiphilium multivorum DSM 11245T	· V · · · L · T · · · · · · · F I · · · · · · · · · · A · V L · · ·
Acidiphilium rubrum	· I · · · L · T F · · · · · · F I · · · · · · · · · · A · V I · · ·
Afifella marina DSM 2698T	· · · · · · A T · · · V · · · V · · · · · · V · · V · I Y · L · · ·
Allochromatium vinosum DSM 180T	· · · · · · · C · · M · · · · I · · · · · · · · · L S · · · L · · ·
Blastochloris sulfoviridis DSM 729T	· V · · · · A · A · · · · Y · V · · · · · · · · · I · L · V G · · ·
Blastochloris viridis DSM 133T	· V · · · · V · M · · · · Y · V · · · · · · · · · I · L · V G · · ·
Blastomonas natatoria DSM 3183T	· · · · · · · T · · M · · · · · I · · · V · · V · I · V · V L · · ·
Bradyrhizobium oligotrophicum DSM 12412T	· · · · · · · C · · · · · M · · I · · · V · · I · V S · · · L · · ·
Bradyrhizobium sp· BTAi1	· · · · · · · C · · · · · M · · I · · · V · · V · V S V · · L · · ·
Caenispirillum salinarum JCM 17360T	· M · M · · · T F · · V · Y · T I T · · · · · · · V · I Y T L · · ·
Cereibacter changlensis DSM 18774T	· S V · · · · · ·
Chloroflexus aggregans DSM 9485T	· I L · · G A T W L C · · · · V Q Y · · V G V · F L · I · V F · Y Y
Chloroflexus aurantiacus DSM 635T	· I L · · G A T W L C · · · · V Q Y · · V G V · F L · I · V F · Y Y
Chloroflexus sp. MS-G	· I L · · G A T W L C · · · · V Q Y · · V G V · F L · I · V F · Y Y
Chloroflexus sp. Y-396-1	· I L · · G A T W L C · · · · V Q Y · · V G V · F L · I · V F · Y Y
Chromatium okenii DSM 169T	· · · · · · · · · · M · · · · I · · · · · · · · · L S · · · I · · ·
Chromatium weissei DSM 5161	· · · · · · · · · · M · · · · I · · · · · · · · · L S · · · I · · ·
Ectothiorhodospira haloalkaliphila ATCC 51935T	· M · V · · · · · · · · · · · V · · · · · · · · · V · I · V L · · ·
Ectothiorhodospira marismortui DSM 4180T	· M · · · · · · · · · · · · · T I · · · · · · · · V · I · · L · · ·
Ectothiorhodospira mobilis DSM 237T	· M · · · · · · · · · · · · · T I · · · · · · · · V · I · V L · · ·
Ectothiorhodospira sp· BSL-9	· V · · · · · · · · · · · · · T · · · · · · V · · V · I · V L · · ·
Ectothiorhodospira sp· PHS-1	· M · V · · · · · · · · · · · V · · · · · · · · · V · I · V L · · ·
Ectothiorhodospira variabilis DSM 21381T	· V · · · · · · · · · · · · · T · · · · · · V · · V · I · V L · · ·
Erythrobacter litoralis DSM 8509T	· S · · · · · C · · · · · Y · F I · · · V · · V · · T V · · I · C ·
Erythrobacter longus DSM 6997T	· · · V · · · C · · · · · Y · F I · · · V · · V · I P I · V L · · ·
Halochromatium glycolicum DSM 11080T	· · · V · · · · · · · · · · · F I · · · · · · · · V · · · · I · · ·
Halochromatium roseum DSM 18859T	· · · V · V · I · · I · · ·
Halochromatium salexigens DSM 4395T	· · · V · V · I · · I · · ·
Halorhodospira abdelmalekii DSM 2110T	· · F · · L A · F · · · · · · F · · · · · · · · · V · A · V L · · ·
Halorhodospira halophila DSM 244T	· · F · · · · T · · · · · · · V · · · · · · · · · V · V · V L · · ·
Halorhodospira halophila IM9622	· · F · · · · T · · · · · · · V I · · · · · · · · V · V · V L · F ·
Halorhodospira halophila Imhoff 9630	· · F · · · · T · · · · · · · V · · · · · · · · · V · V · V L · · ·
Halorhodospira neutriphila DSM 15116T	· S F · · · · T · · · · · · · F I · · · · · · · · V · W · V L · · ·
Ideonella sakaiensis NBRC 110686T	· M · · · · A T F · · · · · · A I · · · · · · · · V · V · · L · · ·
Jannaschia aquimarina DSM 28248T	· S · · · L · T · · · · · · · F I · · · · · · · · V · V · V M · · ·
Lamprobacter modestohalophilus DSM 25653T	· · · V · V · I · · I · · ·
Limnohabitans planktonicus DSM 21594T	· · · · · · A V F · · · · · · I I · · · · · · · · I · V · V M · · ·
Loktanella vestfoldensis DSM 16212T	W · · · · · I T · · · · · Y · · I · · · · · · · · V · · · V C · · Y
Marichromatium bheemlicum DSM 18632T	· · · · · · · C · · M · · · · · · · · · · · · · · L S · · · L · · ·
Marichromatium gracile DSM 203T	· · · · · · · C · · M · · · · · · · · · · · · · · L S · · · L · · ·
Marichromatium purpuratum DSM 1591T	· · · · · · · C · · M · · · · · · · · · · · · · · L S · · · I · · ·
Methylobacterium aquaticum DSM 16371T	· L · M · · · T F · · · · Y · T I · · · · · · · · V P · · V L · · ·
Methylobacterium oryzae DSM 18207T	· L · · · · · T · · M · · · · T I · · · · · V · · V P A · M L · · ·
Methylobacterium platani JCM 14648T	· L · M · · · T F · · · · · · T I · · · · · · · · V P · · V L · · ·
Methylobacterium populi NCIMB 13946T	· M · · · · · T F · · · · Y · T I · · · · · · · · V P · · V L · · ·
Methylobacterium radiotolerans DSM 1819T	· L · · · · · T · · M · · · · T I · · · · · V · · V P V · M L · · ·
Methylobacterium tarhaniae DSM 25844T	· L · M · · · T F · · · · Y · T I · · · · · · · · V P · · V L · · ·
Methylobacterium variabile DSM 16961T	· L · · · · · T F · · · · Y · T I · · · · · · · · V P V · V L · · ·
Nereida ignava DSM 16309T	· S · · · · · T · · · · · · · F I · · · · · · · · V F V · M M · · ·
Niveispirillum cyanobacteriorum LMG 28334T	· V · · · · · T · · · · · · · I I · · · · · · · · V · · · · L · · ·
Novosphingobium fuchskuhlense DSM 25065T	· · · · · · · T · · · · · · · · I · · · · · · · · V F I · · L · A ·
Novosphingobium subterraneum DSM 12447T	· · · · · · · T · · · · · · · · I · · · V · · V · V · V · M I · · ·
Oceanibaculum indicum LMG 24626T	· V · · · · · T · · · · · · · I I · · · · · · · · I · · · · I · · ·
Pararhodospirillum photometricum DSM 122T	· V · V · · · C M · M I · · · V · · · · · · · · · M · · · · L · · ·
Phaeospirillum fulvum MGU-K5	· M · V · · · · · · M I · · · A · · · · · · · · · V S · · · L · · ·
Phaeospirillum molischianum DSM 120T	· M · V · · · · · · M I · · · A · · · · · · · · · V S · · · L · · ·
Polynucleobacter duraquae DSM 21495T	· · · · · L · T · · · · · · · I I · · · V · · · · V P L · M M · · ·
Porphyrobacter neustonensis DSM 9434T	· · · V · · · C · · · · · Y · F I · · · V · · V · I P I · V L · · ·
Porphyrobacter sanguineus DSM 11032T	· · · V · · · C · · · · · Y · F I · · · V · · V · I P I · V L · · ·
Prosthecomicrobium hirschii ATCC 27832T	· V · · · · · T F · · · · · · T I · · · · · · · · V · · · V L · · ·
Pseudohaliea rubra DSM 19751T	· · · · · · · T · · M · · · · D · · · · · · · · · L · · Y V L · · ·
Rhodobacter azotoformans IFO 16436T	· V ·
Rhodobacter blasticus DSM 2131T	· ·
Rhodobacter capsulatus DSM 1710	· V · · · L · · W · M · · Y · · M · · · V · · · · M · V · M I · · ·

	L181	M133	M210	M214	M252	L179	L182	L184	L185	L186	L189	L193	L212	L215	L216	L219	L220	L221	L222	L223	L224	L225	L229	L232	L236	M146	M147	L119	L121	L123	M127	M129	M130	M148
Cereibacter sphaeroides 2.4.1	F	T	Y	L	W	F	T	A	L	A	L	L	E	F	F	L	V	G	Y	S	I	G	I	L	L	T	A	F	F	F	W	W	W	W
Rhodobacter megalophilus DSM 18937T	·	·	·	·	·	·	·	·	·	·	·	·	·	·	·	·	·	·	·	·	·	·	·	·	·	·	·	·	·	·	·	·	·	·
Rhodobacter veldkampii DSM 11550T	·	·	·	M	·	L	·	T	·	·	·	·	·	·	·	·	·	·	·	·	V	·	·	·	·	·	·	·	·	·	L	·	·	·
Rhodoblastus acidophilus DSM 137T	·	V	·	·	·	·	·	C	·	·	M	V	·	·	·	V	·	·	·	·	·	·	·	·	·	M	·	·	·	·	L	·	·	·
Rhodocista centenaria ATCC 51521	·	·	·	·	·	·	A	T	F	·	·	·	·	·	Y	T	I	·	·	·	·	·	·	I	·	·	·	I	Y	V	L	·	·	·
Rhodocyclus tenuis Imhoff 230	·	·	·	·	·	·	·	T	·	·	·	·	·	·	·	V	·	·	·	·	·	·	·	·	·	V	·	V	·	·	L	F	·	·
Rhodoferax fermentans	·	I	·	·	·	·	G	T	F	·	·	·	·	·	·	I	I	·	·	·	·	·	·	·	·	V	·	·	·	V	I	·	·	·
Rhodoplanes elegans DSM 11907T	·	V	·	V	·	·	·	·	·	·	M	·	·	I	·	T	·	·	·	·	·	·	·	·	·	L	·	L	·	·	L	·	·	·
Rhodopseudomonas faecalis JCM 11668T	·	·	·	·	·	·	·	C	·	·	·	·	·	V	·	·	·	·	·	·	·	·	·	V	·	V	S	V	·	·	L	·	·	·
Rhodopseudomonas palustris DSM 126	·	·	·	·	·	·	·	C	·	·	·	·	·	V	·	·	·	·	·	·	·	·	·	·	·	V	S	·	·	·	A	·	·	·
Rhodopseudomonas pseudopalustris DSM 123T	·	·	·	·	·	·	·	C	·	·	·	·	·	I	·	·	·	·	·	·	·	·	·	·	·	V	S	·	·	·	A	·	·	·
Rhodopseudomonas rhenobacensis DSM 12706T	·	·	·	·	·	·	·	C	·	·	·	·	·	V	·	·	·	·	·	·	·	·	·	V	I	V	S	·	·	·	V	·	·	·
Rhodospira trueperi ATCC 700224T	·	·	·	·	·	·	V	C	F	·	·	·	·	·	·	F	I	·	·	·	·	·	·	·	·	V	·	I	·	V	G	·	·	·
Rhodospirillum rubrum DSM 107	·	M	·	·	·	·	·	C	·	·	·	·	·	Y	·	T	I	·	·	·	V	·	·	V	·	A	·	·	·	·	L	·	·	·
Rhodospirillum rubrum DSM 467T	·	M	·	·	·	L	·	C	·	·	·	·	·	Y	·	T	I	·	·	·	V	·	·	V	·	·	·	·	·	·	L	·	·	·
Rhodospirillum rubrum F11	·	M	·	·	·	L	·	C	·	·	·	·	·	Y	·	T	I	·	·	·	V	·	·	V	·	·	·	·	·	·	L	·	·	·
Rhodospirllum rubrum DSM 1068	·	M	·	·	·	·	·	C	·	·	·	·	·	Y	·	T	I	·	·	·	V	·	·	V	·	A	·	·	·	·	L	·	·	·
Rhodothalassium salexigens DSM 2132T	·	S	·	·	·	·	A	T	F	·	·	A	·	·	·	F	M	·	·	·	·	·	·	·	·	V	·	I	·	V	I	·	·	·
Rhodovibrio salinarum DSM 9154T	·	·	·	·	·	·	·	T	F	·	·	A	·	·	·	F	I	·	·	·	·	·	·	·	·	V	·	·	·	V	L	·	·	·
Rhodovibrio sodomensis DSM 9895T	·	·	·	·	·	·	·	T	·	·	·	A	·	·	·	F	I	·	·	·	·	·	·	·	·	V	·	·	·	V	L	·	·	·
Rhodovulum adriaticum DSM 2781T	·	·	·	·	·	L	·	C	·	·	·	·	·	Y	·	·	I	·	·	·	·	·	·	I	·	I	·	I	·	V	·	·	·	·
Rhodovulum euryhalinum DSM 4868T	·	·	·	·	·	L	A	C	·	·	·	·	·	·	·	·	I	·	·	·	·	·	·	·	·	I	·	I	·	V	·	·	·	·
Rhodovulum sulfidophilum DSM 1374T	·	·	·	·	·	L	A	C	·	·	·	·	·	·	·	·	I	·	·	·	·	·	·	A	·	I	·	I	·	M	·	·	·	·
Rhodovulum visakhapatnamense DSM 17937T	·	·	·	·	·	L	A	C	·	·	·	·	·	·	·	·	I	·	·	·	·	·	·	A	·	I	·	I	·	V	·	·	·	·
Roseateles depolymerans DSM 11813T	·	M	·	·	·	·	A	T	F	·	·	·	·	·	·	A	I	·	·	·	·	·	·	·	·	V	·	·	·	V	L	·	·	·
Roseateles terrae CCUG 52222T	·	M	·	·	·	·	A	T	F	·	·	·	·	·	·	A	I	·	·	·	·	·	·	·	·	V	·	·	·	V	L	·	·	·
Roseibacterium elongatum DSM 19469T	·	·	·	·	·	L	·	T	·	·	·	·	·	·	·	F	I	·	·	·	·	·	·	·	·	I	·	·	·	·	M	·	·	·
Roseisalinus antarcticus DSM 11466T	·	·	·	·	·	·	·	C	F	·	·	·	·	Y	·	·	I	·	·	·	·	·	·	·	·	V	·	·	·	V	V	·	·	·
Roseivivax halodurans DSM 15395T	·	S	·	·	·	·	·	T	F	·	·	·	·	·	·	F	I	·	·	·	V	·	·	I	·	V	F	·	·	·	M	·	·	·
Roseivivax halotolerans DSM 15490T	·	S	·	·	·	L	·	T	·	·	·	·	·	·	·	F	I	·	·	·	V	·	·	·	·	V	F	I	·	·	M	·	·	·
Roseivivax isoporae DSM 22223T	·	S	·	·	·	L	A	T	·	·	·	·	·	·	·	F	I	·	·	·	V	·	·	V	·	V	F	·	·	·	M	·	·	·
Roseobacter denitrificans DSM 7001T	·	A	·	·	·	L	·	T	·	·	·	·	·	·	·	F	I	·	·	·	V	·	·	·	·	I	F	·	·	A	M	·	L	·
Roseococcus thiosulfatophilus DSM 8511T	L	·	·	·	·	·	·	T	·	·	·	·	·	·	Y	F	I	·	·	·	·	·	·	·	I	·	F	W	Y	M	L	·	L	·
Roseovarius indicus LMG 24622T	·	·	·	·	·	L	·	C	·	·	·	·	·	·	·	·	I	·	·	·	·	·	·	·	·	V	·	·	·	·	C	·	·	·
Roseovarius mucosus DSM 17069T	·	·	·	·	·	L	·	T	F	·	·	·	·	·	·	F	I	·	·	·	V	·	·	V	·	V	·	·	·	·	C	·	·	·
Roseovarius tolerans DSM 11457T	·	S	·	·	·	·	·	T	·	·	·	·	·	·	·	F	I	·	·	·	V	·	·	V	·	V	F	·	·	V	L	·	·	·
Rubritepida flocculans DSM 14296T	·	V	·	·	·	·	·	T	·	·	·	·	·	·	Y	F	I	·	·	·	·	·	·	·	I	·	F	W	Y	M	F	·	A	·
Rubrivivax benzoatilyticus ATCC BAA-35T	·	M	·	·	·	·	·	T	·	·	M	·	·	·	·	A	·	·	·	·	·	·	·	·	·	V	·	I	·	·	L	·	·	·
Rubrivivax gelatinosus DSM 149	·	M	·	·	·	·	·	T	·	·	M	·	·	·	·	A	·	·	·	·	·	·	·	·	·	V	·	I	·	·	L	·	·	·
Rubrivivax gelatinosus DSM 1709T	·	M	·	·	·	·	·	T	·	·	M	·	·	·	·	A	·	·	·	·	·	·	·	·	·	V	·	I	·	·	L	·	·	·
Rubrivivax gelatinosus IL144	·	M	·	·	·	·	·	T	·	·	M	·	·	·	·	A	·	·	·	·	·	·	·	·	·	V	·	I	·	·	L	·	·	·
Rubrivivax gelatinosus IM 151	·	M	·	·	·	·	·	T	·	·	M	·	·	·	·	A	·	·	·	·	·	·	·	·	·	V	·	I	·	·	L	·	·	·
Rubrivivax gelatinosus IM 456	·	M	·	·	·	·	·	T	·	·	M	·	·	·	·	A	·	·	·	·	·	·	·	·	·	V	·	I	·	·	L	·	·	·
Salipiger mucosus DSM 16094T	·	·	·	·	·	L	A	C	·	·	·	·	·	·	·	F	I	·	·	·	·	·	·	·	·	V	F	·	·	·	M	·	·	·
Skermanella aerolata DSM 18479T	·	·	·	·	·	·	·	T	F	·	·	·	·	·	·	T	I	·	W	·	·	·	·	·	·	V	S	V	·	V	M	·	·	·
Skermanella stibiiresistens SB22T	·	·	·	·	·	·	·	T	F	·	·	·	·	·	·	T	I	·	W	·	·	·	·	·	·	V	S	V	·	·	M	·	·	·
Sphingomonas sanxanigenens DSM 19645T	·	·	·	·	·	·	V	G	G	·	F	·	·	·	·	T	I	·	·	·	V	·	·	·	·	V	·	·	Y	·	I	·	·	·
Sulfitobacter guttiformis DSM 11458T	·	·	·	·	·	·	·	C	·	·	·	·	·	Y	·	·	I	·	·	·	·	·	·	·	·	V	·	V	·	V	I	·	·	·
Sulfitobacter noctilucicola DSM 101015T	·	·	·	·	·	·	·	C	·	·	·	·	·	Y	·	·	I	·	·	·	·	·	·	·	·	V	·	V	·	V	I	·	·	·
Synechocystis sp· PCC 6803	·	Q	L	·	·	G	G	S	·	F	M	·	A	Y	·	I	F	·	·	·	N	N	L	F	W	N	·	V	Y	A	I	F	M	I
Thalassobacter stenotrophicus CECT5294T	·	·	·	·	·	·	·	T	·	·	·	·	·	·	·	F	I	·	·	·	V	·	·	V	·	V	·	V	·	·	S	·	·	·
Thiocapsa bogorovi BBS	·	·	·	·	·	·	·	T	·	·	M	·	·	·	·	·	I	·	·	·	·	·	·	·	·	L	S	·	·	·	I	·	·	·
Thiocapsa rosea DSM 235T	·	·	·	·	·	·	·	C	·	·	M	·	·	·	·	·	I	·	·	·	·	·	·	·	·	L	S	·	·	·	I	·	·	·
Thiocapsa roseopersicina DSM 217T	·	·	·	·	·	·	·	C	·	·	M	·	·	·	·	·	I	·	·	·	V	·	·	·	·	L	S	·	·	·	I	·	·	·
Thiococcus pfennigii DSM 228	·	V	·	·	·	·	A	·	F	·	·	I	·	·	·	V	L	·	·	·	·	·	·	·	·	V	·	I	·	·	L	·	·	·
Thiococcus pfennigii Pfennig 8320	·	V	·	·	·	·	A	·	F	·	·	I	·	·	·	V	L	·	·	·	·	·	·	·	·	V	·	I	·	·	L	·	·	·
Thiocystis minor DSM 178T	·	·	·	·	·	·	·	·	·	·	M	·	·	·	·	I	·	·	·	·	·	·	·	·	·	L	S	·	·	·	L	·	·	·
Thiocystis violacea DSM 207T	·	·	·	·	·	·	·	C	·	·	M	·	·	·	·	I	·	·	·	·	·	·	·	·	·	L	S	·	·	·	I	·	·	·
Thiocystis violacea DSM 208	·	·	·	·	·	·	·	C	·	·	M	·	·	·	·	I	·	·	·	·	·	·	·	·	·	L	S	·	·	·	L	·	·	·
Thiocystis violascens DSM 198T	·	·	·	·	·	·	·	·	·	·	M	·	·	·	·	I	·	·	·	·	·	·	·	·	·	L	S	·	·	·	L	·	·	·
Thioflavicoccus mobilis ATCC 700959T	·	V	·	·	·	·	A	·	F	·	·	I	·	·	·	V	L	·	·	·	V	·	·	·	·	V	·	I	·	·	L	·	·	·
Thiohalocapsa halophila DSM 6210T	·	·	·	V	·	·	·	·	·	·	·	·	·	·	·	F	I	·	·	·	·	·	·	·	·	V	·	·	·	·	I	·	·	·
Thiorhodococcus drewsii DSM 15006T	·	·	·	·	·	·	·	C	·	·	M	·	·	·	·	I	·	·	·	·	·	·	·	·	·	L	S	·	·	·	I	·	·	·
Thiorhodococcus mannitoliphagus DSM 18266T	·	·	·	·	·	·	·	C	·	·	M	·	·	·	·	I	·	·	·	·	·	·	·	·	·	L	S	·	·	·	L	·	·	·
Thiorhodococcus minor DSM 11518T	·	·	·	·	·	·	·	C	·	·	M	·	·	·	·	I	·	·	·	·	·	·	·	·	·	L	S	·	·	·	L	·	·	·
Thiorhodospira sibirica ATCC 700588T	·	·	F	·	·	·	·	·	·	·	·	·	·	·	·	V	·	·	·	·	·	·	·	·	·	V	·	A	·	V	L	·	·	·
Thiorhodovibrio winogradskyi DSM 6702T	·	V	·	V	·	·	·	C	·	·	M	·	·	V	·	I	·	·	·	·	·	·	·	·	·	L	·	·	·	·	L	·	·	·

Supplementary Table 1 | Mutant RCs of the Trp scanning series.

Mutant #	Native residue	Distance from H_B, Å[a]	Distance from Q_B, Å[a]	Orientation of side chain relative to axis of interaction[b]	Closest atoms to axis, Å[a]	$C_α$ distance to axis, Å[a]	C_2 symmetry-related residue	RC yield (%WT)[c]	$P^+Q_B^-$ Yield (%WT)[d]
S34[e]	YEFHV parent	---	---	-	---	---	---	20.8	27.6
S103	PheL216	4.3	3.4	Toward	2.8	6.6	TrpM252	15.4	36.8
S86	PheL179	8.2	13.3	Away from	11.4	12.6	LeuM209	1.1	28.8
S82	LeuL185	3.1	6.5	Toward	1.3	4.3	LeuM214	46.4	26.6
S99	AlaM147	5.7	11.5	Away from	9.8	10.3	ProL118	3.7	24.5
S113	LeuL236	9.8	8.6	Toward	8.8	12.6	MetM272	8.3	28.8
S85	ThrL182	5.1	9.1	Perpendicular	6.6	7.5	GlyM211	41.1	23.6
S105	ValL220	3.8	6.7	Toward	6.8	8.9	MetM256	8.0	20.7
S112	LeuL232	10.1	5.3	Toward	6.9	9.5	TrpM268	12.8	20.1
S107	TyrL222	8.1	4.9	Perpendicular	6.7	8.9	PheM258	5.6	18.1
S93	AlaL186	6.0	4.6	Perpendicular	4.6	4.6	LeuM215	1.3	19.0
S106	GlyL221	8.5	8.8	Away from	10.4	11.4	GlyM257	24.7	14.6
S101	GluL212	11.4	3.3	Perpendicular	6.0	6.7	AlaM248	25.1	9.2
S109	IleL224	10.7	3.0	Away from (turn)	4.6	5.9	AlaM260	3.6	8.7
S102	PheL215	6.4	7.4	Perpendicular	7.2	8.9	PheM251	33.9	7.0
S91	LeuL189	3.6	3.5	Toward	0.2	3.4	MetM218	14.1	7.2
S104	LeuL219	3.3	7.8	Toward & perpendicular	6.7	9.7	ThrM255	18.3	5.2
S84	AlaL184	3.5	9.3	Away from	6.5	8.0	AlaM213	3.4	5.0
S87	LeuL193	8.3	3.5	Toward	3.5	6.3	ThrM222	17.5	4.6
S110	GlyL225	12.7	2.9	Away from (turn) & perpendicular	4.7	5.3	ThrM261	36.2	4.5
S108	SerL223	10.3	3.2	Toward	5.4	6.0	AsnM259	17.6	4.0
S98	ThrM146	3.6	7.3	Toward	6.7	8.1	IleL117	1.8	3.4
S111	IleL229	10.0	3.5	Toward	4.0	5.5	IleM265	6.5	2.5
S83	AlaL192	5.9	6.6	Perpendicular	7.4	8.0	AlaM221	0.4	ND[g]
S88	AlaL188	4.0	6.3	Away from	5.5	6.2	AlaM217	0.5	ND
S89	AsnL183	5.9	9.2	Away from	8.0	9.0	SerM212	0.4	ND
S90	HisL190	7.4	2.8	Perpendicular	4.2	4.7	HisM219	0.3	ND
S92	GlyL191	8.5	6.0	Away from	6.8	8.1	GlyM220	0.4	ND
S94	ValL194	11.2	3.6	Perpendicular	6.8	7.9	IleM223	0.3	ND
S95	LeuL187	7.2	6.8	Away from	6.3	7.3	PheM216	0.6	ND
S96	ValM274	4.0	12.8	Perpendicular	10.3	10.5	LeuL238	0.9	ND
S97	AlaM273	3.5	11.0	Toward	9.2	10.4	SerL237	1.5	ND
S100	AlaM149	3.5	10.1	Toward	7.2	8.5	AlaL120	1.2	ND
S162[f]	PheM150	3.4	11.7	X (H_B between)	4.9	6.4	PheL121	8.0	ND

[a] Distances between the individual atom on each entity that was closest to any atom of the other molecule (not including H or phytyl tail atoms of either H_B or Q_B), except for distances measured between the α carbon of the residue and the axis of interaction. The axis (Fig. 3a) is defined as a line drawn from the center of the H_B ring to the center of the Q_B ring. Measured in PyMOL using the WT RC structure (PDB code: 1aig).

[b] Orientations reference the native RC structure only.

[c] RC expression yields were estimated in 80-ml cultures grown in ^{G}YCC medium in baffled 125-ml Nephelo flasks. Cells were ruptured mechanically, cell debris was removed, and the UV-vis-nearIR spectrum of the cleared lysate was recorded at room temperature with a Shimadzu UV-1800 spectrophotometer. The area of the ~865nm peak was used to quantify RC expression, following correction of the baseline using a customized method. Expression yields for mutant RCs were related to the yield for WT RCs (%WT).

[d] Residues listed in order of descending $P^+Q_B^-$ yield.

[e] Parent *C. sphaeroides* RC carrying the YEFHV substitutions (Fig. 1d) to which each Trp substitution was added.

[f] Purified RCs were structurally unstable.

[g] Not determined

Supplementary Table 2 | Mutant RCs of the Trp cluster† near H_B.

Mutant #	Site(s) of Phe Substitution$^{\Delta}$	Distance(s) from $H_B$$^{\pm}$	C_2 Symmetry-Related Residue(s)	RC Expression Yield (% WT)§	$P^+Q_B^-$ Yield (% WT)¥
S114	M127	11.4	ValL98	20.8	26.0
S115	M129	2.9	TrpL100	14.7	15.0
S116	M130	6.8	AlaL101	11.0	22.3
S117	M127, M129	11.4, 2.9	ValL98, TrpL100	3.3	15.9
S118	M129, M130	2.9, 6.8	TrpL100, AlaL101	1.6	13.2
S119	M127, M130	11.4, 6.8	ValL98, AlaL101	2.9	23.1
S120	M127, M129, M130	11.4, 2.9, 6.8	ValL98, TrpL100, AlaL101	1.3	ND€
S161	M148	9.6	PheL119	28.9	25.0

† In the RC structure, a cluster of tryptophan residues is located near H_B of the inactive branch of cofactors, while a similar region near H_A is rich in phenylalanine residues (Fig. 2a,b). To more closely emulate the environment of H_A, the residues within the tryptophan cluster near H_B were targeted for substitution with Phe, both individually and in a combinatorial manner. The consequences of these substitutions on the ability of B-side ET to form $P^+Q_B^-$ were determined.

$^{\Delta}$ All mutations were constructed in the YEFHV background (Fig. 1d).

± Distances reported are between the closest atom on the aromatic ring of the native tryptophan residue and the closest atom on the macrocycle ring of H_B.

§ RC expression yield determined as described for in Methods.

€ ND = not determined

¥ Yields of state $P^+Q_B^-$ formed via the activity of the B-path cofactors determined with the millisecond assay as described in the methods.

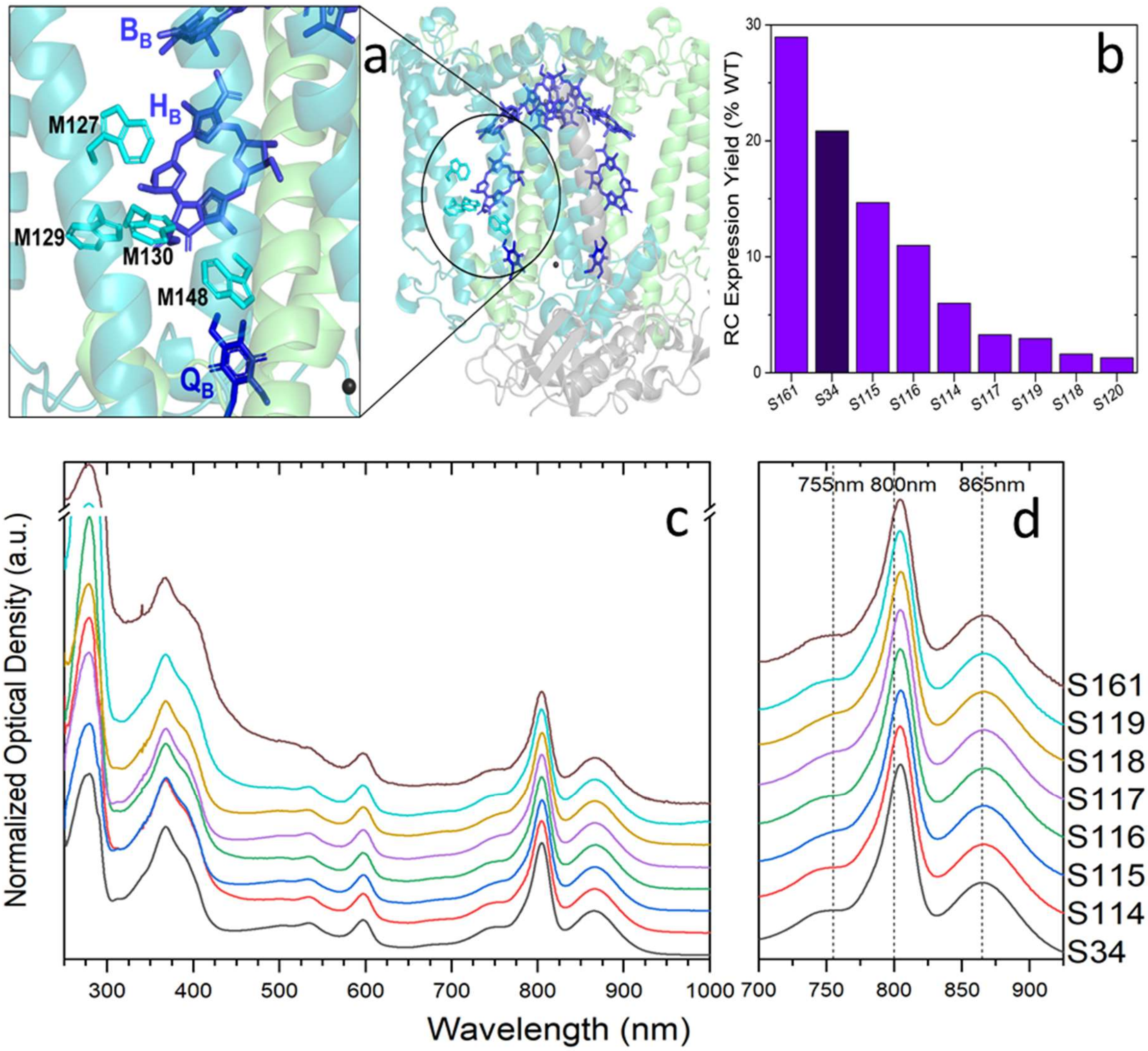

Supplementary Figure 2 | RC expression yields, ground-state spectra – Trp Cluster near H_B

a, Sites of residues of the Trp cluster near H_B that were substituted with Phe, both singly and in combination. **b,** Expression yields for mutants of the Phe cluster near H_A as described for Figure S1. **c,** Ground state UV-vis-nearIR spectra from 250-1000 nm of Trp cluster mutant RCs recorded at RT. **d,** Spectral region 700-950 nm focusing on the Q_y transitions of the cofactors. Spectra were normalized to a constant value for P_{max} near ~865 nm and were offset by a defined value for overlay. Several substitutions of the Trp cluster residues with Phe negatively impacted the expression yield (**b**) and thus the purity of RCs prepared from some strains in this series, as evident from the large signal near ~280 nm (**c**). Dashed lines at 865 nm and 800 nm (**d**) Q_y maxima attributable to P and B_A/β_A/B_B, respectively, and the positions of these maxima are essentially the same in all mutants and the parent RC (S34; YEFHV). Because of the proximity of Trp residues to H_B (**a**), the substitution with Phe slightly alters the maximum position of the broad absorption near 755 nm that is assigned to H_B. Phe substitutions of the Trp cluster have slight, if any, effects on the Q_x transitions of the tetrapyrrole cofactors.

Supplementary Table 3 | Mutant RCs of the Phe Cluster[€] near H_A.

Mutant #	Background Substitution(s)[†]	Site(s) of Trp Substitution	C_2 Symmetry-Related Residue(s)	RC Expression Yield (% WT)[¥]
S75	V	None	---	39
S128	V	L119	TrpM148	68
S129	V	L121	PheM150	66
S130	V	L123	SerM152	55
S131	V	L119,L121	TrpM148, PheM150	54
S132	V	L119,L123	TrpM148, SerM152	49
S133	V	L121,123	PheM150, SerM152	48
S134	V	L119,L121,L123	TrpM148, PheM150, SerM152	48
S137	None	L119	TrpM148	61
S138	None	L121	PheM150	25
S139	None	L123	SerM152	42
S140	None	L119,L121	TrpM148, PheM150	56
S141	None	L119,L123	TrpM148, SerM152	63
S142	None	L121,123	PheM150, SerM152	31
S143	None	L119,L121,L123	TrpM148, PheM150, SerM152	65
S78	HV	None	---	35
S144	HV	L119	TrpM148	40
S145	HV	L121	PheM150	39
S146	HV	L123	SerM152	36
S147	HV	L119,L121	TrpM148, PheM150	40
S148	HV	L119,L123	TrpM148, SerM152	37
S149	HV	L121,123	PheM150, SerM152	35
S150	HV	L119,L121,L123	TrpM148, PheM150, SerM152	6

[€]In the RC structure, a cluster of phenylalanine residues is located near H_A of the active ET pathway, while a similar area near H_B is rich in tryptophan residues (Fig. 2a,b). To more closely emulate the environment of H_B, the residues within the phenylalanine cluster near H_A were targeted for substitution with Trp, both individually and in a combinatorial manner. [†]V = ValM252; HV = HisM214-ValM252. [¥]RC expression yields were determined as described in Methods.

Supplementary Table 4 | Effects of Phe cluster mutants on P* Lifetime[§] in the ValM252 background.

Mutant #	Sample	τ_1 (ps)	A_1	τ_2 (ps)	A_2	τ_{avg}[¥] (ps)
S75	W(M252)V	2.8	0.85	13.4	0.15	4.4
S128	F(L119)W+V	3.0	0.83	14.5	0.17	4.9
S129	F(L121)W+V	3.0	0.78	15.0	0.22	5.6
S130	F(L123)W+V	2.9	0.83	14.0	0.17	4.7
S131	F(L119,L121)W+V	3.2	0.79	17.3	0.21	6.2
S132	F(L119,L123)W+V	2.7	0.81	12.9	0.19	4.7
S133	F(L121,L123)W+V	3.1	0.78	16.9	0.22	6.1
S134	F(L119,L121,L123)W+V	3.1	0.80	18.0	0.20	6.1

[§]From decay of the stimulated emission at 920 nm

[¥]Amplitude-weighted average lifetime.

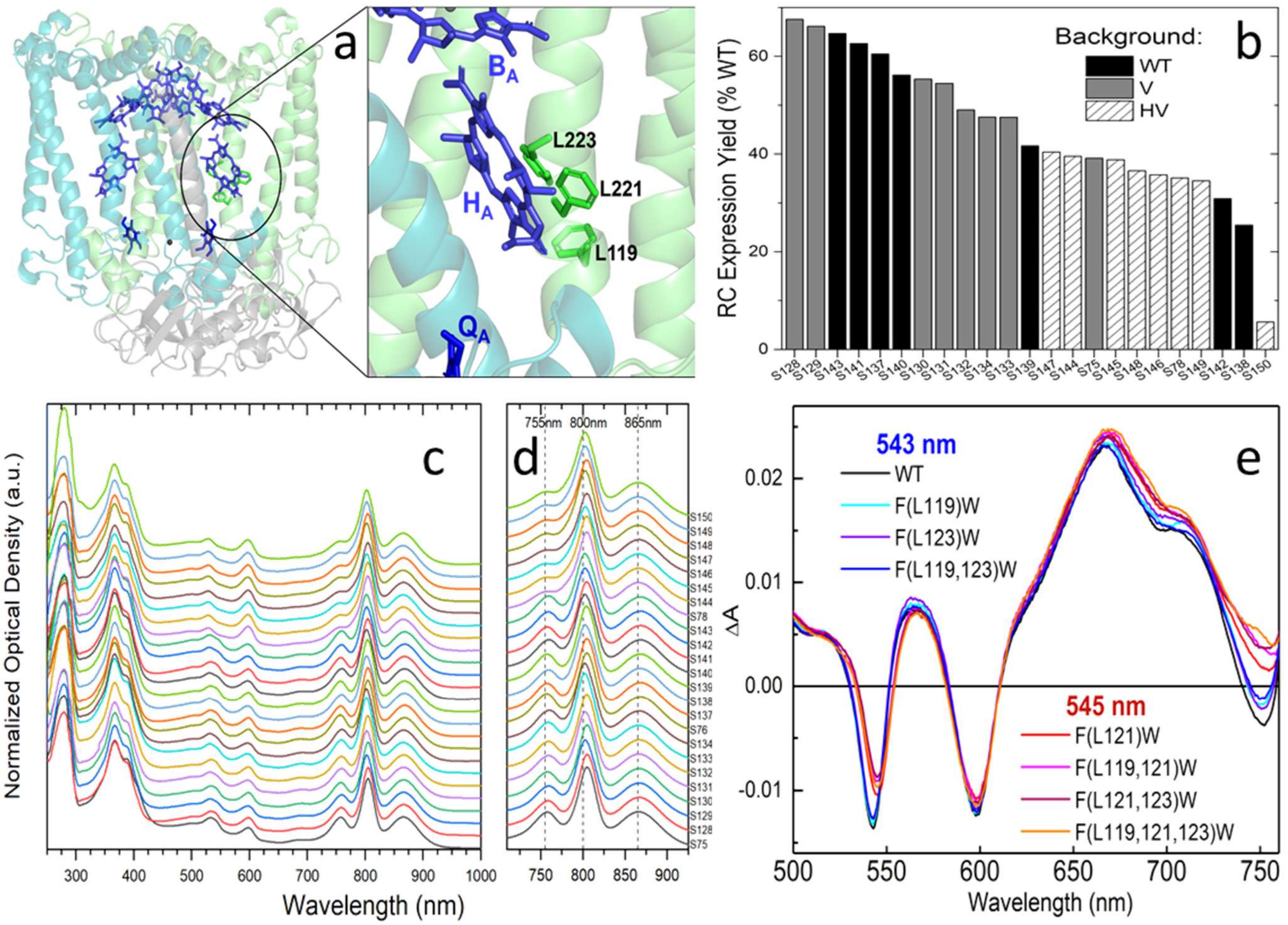

Supplementary Figure 3 | RC expression yields, ground-state spectra, ultrafast-transient spectra – Phe Cluster near H_A. **a,** Sites of residues of the Phe cluster near H_A that were substituted singly and in combination with Trp. **b,** Expression yields for mutants of the Phe cluster near H_A as described in Methods. **c,** Ground state spectra recorded at RT. **d,** Spectral region from 700-950 nm focusing on the Q_y transitions of the B_A/β_A/H_B and P cofactors. Spectra were normalized to a constant value for P_{max} near ~865 nm and were offset by a defined value for overlay. The location of the maximum for the P transition varies only slightly from the value of 865 nm (dashed line, part d) seen in the parent RCs (S75, S76, S78). The presence of the F(L121)W mutation is linked to ~2-3 nm blue shifts in the Q_y peak near ~800 nm (dashed line; part d) that is assigned to Bchl monomers B_A, B_B, and β_A. Shifts are most prominent in the V background, followed by the WT, and are lesser in the HV background. The presence of Trp at L121 also causes slight red shifts of ~1-3 nm in the Q_y transitions assigned to H_A and H_B in the 760 nm region; these red shifts are observed in mutants constructed in the H_A-containing V and WT backgrounds only. These spectral shifts are linked directly to the Trp substitution as previous replacement of PheL121 by Leu had no spectral effects. Trp substitutions of residues of the Phe cluster have slight, if any, effects on the Q_x transitions of H_A and H_B in the 525-535 nm region. **e,** Time-resolved difference spectra for state $P^+H_A^-$ at the time of maximum amplitude for mutant RCs of the Phe cluster near H_A in the Q_x spectral region. The negative feature at 543 or 545 nm is bleaching of the Q_x band of H_A, the negative feature at ~600 is bleaching of the Q_x band of P, and the positive feature at ~655 nm is the H_A anion.

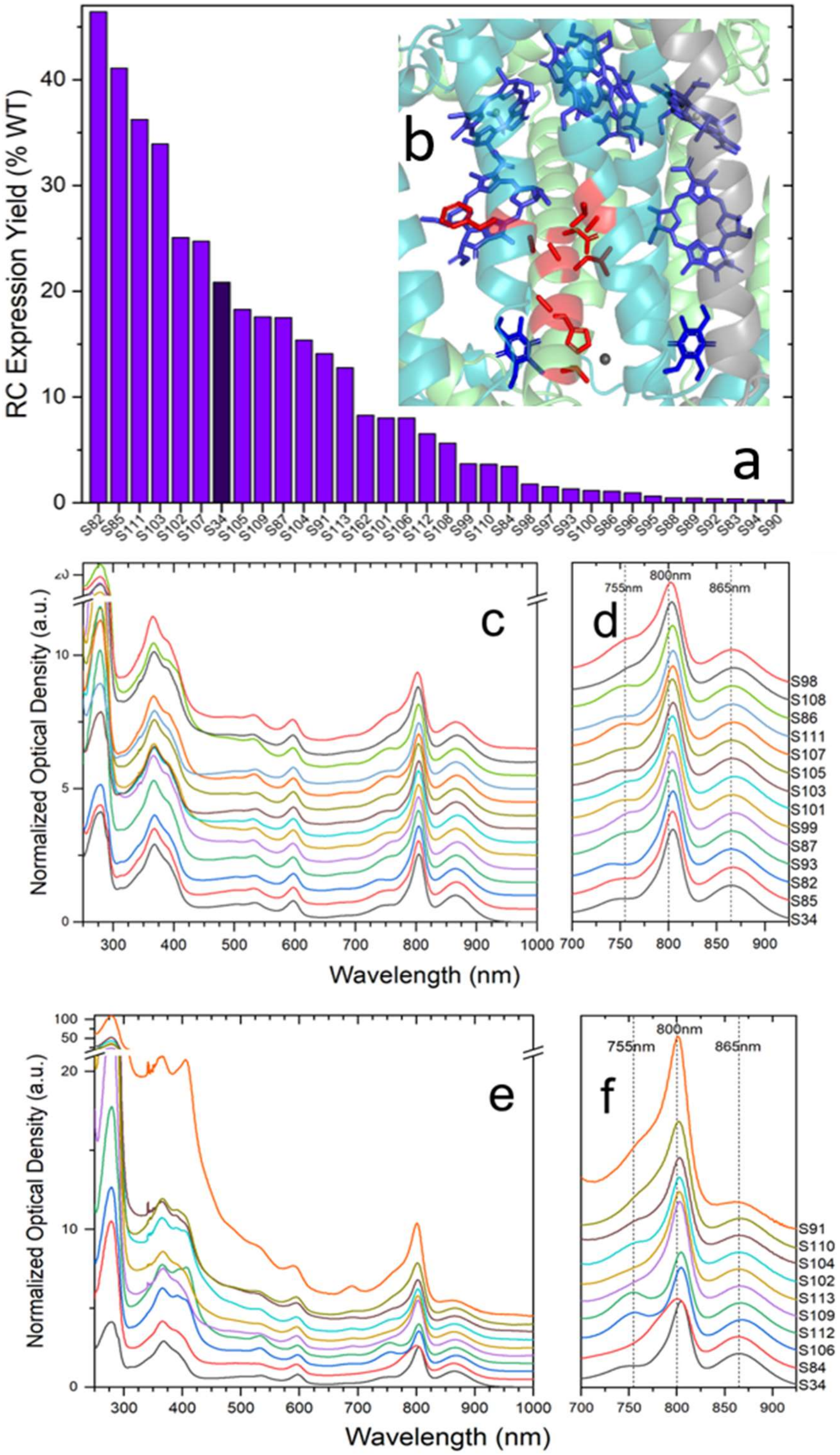

Supplementary Figure 4 | RC expression yields, ground-state spectra. a, RC expression yields for the Trp-scanning series. **b,** Axis of poor expression yield. Bulky Trp substitutions that have a negative impact on folding and assembly of the protein-cofactor complex cluster at sites within a portion of the L-M heterodimer interface involving the interior surfaces of the D helix of the L subunit (green) and the E helix of the M subunit (cyan). Other sites of disruptive Trp substitutions lie on the exterior face of the C helix of the M subunit that interacts with H_B or the residues on the B helix of the M chain. Phytyl tails of cofactors are removed for visual clarity. H subunit – grey; PDB code: 1aig. **c, e,** Ground state UV-vis-nearIR spectra from 250-1000 nm of subsets of purified mutant RCs of the Trp-scanning series recorded at room temperature. **d,f,** Spectral region from 700-950 nm focusing on the Q_y transitions of the cofactors. Spectra were normalized to a constant value for P_{max} near ~865 nm and were offset by a defined value for overlay. As evident from the large signal near ~280 nm (**c**), substitutions of the native residue by Trp negatively impacted the expression yield and thus the purity of RCs prepared from some strains in this series. Dashed lines at 865 nm and 800 nm (**d,f**) identify absorptions of P and $B_A/\beta_A/B_B$, respectively; the positions of these maxima in all mutants show only slight shifts, if any, in comparison to the spectrum of the parent RC (S34; YEFHV). Because of the proximity of many of the sites to H_B, substitution of the native residue by Trp changes the maximum position of the broad absorption near 755 nm that is assigned to H_B (**d,f**). Substitutions in the Trp-scanning series have slight, if any, effects on the Q_x transitions of the tetrapyrrole cofactors.

Supplementary Table 5 | RCs carrying combinations of substitutions.

Mutant #	Mutation(s)	$P^+Q_B^-$ yield (% WT)
	**Substitutions added to the YEFHV background**	
S34	YEFHV Parent	27.6
S186	+L(L185)W	n.d.
S103	+F(L216)W	n.d.
S205	+L(L185)W, F(L216)W	42.3
S189	+L(L185)R, F(L216)W	n.d.
S234	+L(L193)T	54.9
S235	+L(L185)W, L(L193)T	51.1
S217	+L(L193)T, F(L216)W	62.4
S218	+L(L193)S, F(L216)W	39.3
S221	+L(L185)W, L(L193)T, F(L216)W	65.2
S222	+L(L185)W, L(L193)S, F(L216)W	36.2
S219	+L(L185)R, L(L193)T, F(L216)W	n.d.
S220	+L(L185)R, L(L193)S, F(L216)W	n.d.
	**Substitutions added to the YFHV background**	
S54	YFHV parent	6.3
S210	+L(L185)W	22.9
S151	+F(L216)W	6.7
S216	+L(L185)W, F(L216)W	23.6
S236	+L(L193)T	13.7
S237	+L(L185)W, L(L193)T	33.5
S238	+L(L193)T, F(L216)W	9.4
S239	+L(L185)W, L(L193)T, F(L216)W	34.6

Supplementary Table 6 | Identity of additional mutants in the Trp-scan series and RCs carrying combinations of Trp-scan, Phe-cluster, and/or Trp-cluster substitutions.

Mutant #	Mutation(s)	$P^+Q_B^-$ yield (% WT)
	**Substitutions added to the YEFHV background**	
S82	+L(L185)W	26.6
S83	+L(L192)W	n.d.
S84	+A(L184)W	5.0
S85	+T(L182)W	23.6
S86	+F(L179)W	28.8
S87	+L(L193)W	4.6
S88	+A(L188)W	n.d.
S89	+N(L183)W	n.d.
S90	+H(L190)W	n.d.
S91	+L(L189)W	7.2
S92	+G(L191)W	n.d.
S93	+A(L186)W	19.0
S94	+V(L194)W	n.d.
S95	+L(L187)W	n.d.
S97	+(M274)W	n.d.
S98	+T(M146)W	3.4
S99	+A(M147)W	24.5
S100	+A(M149)W	n.d.
S101	+E(L212)W	9.2
S102	+F(L215)W	7.0
S105	+V(L220)W	20.7
S106	+G(L221)W	14.6
S107	+Y(L222)W	18.1
S108	+S(L223)W	4.0
S109	+I(L224)W	8.7
S110	+G(L225)W	4.5
S111	+I(L229)W	2.5
S112	+L(L232)W	20.1
S113	+L(L236)W	28.8
S121	+F(L216)W, W(M127)F	33.6
S122	+F(L216)W, W(M129)F	23.0
S123	+F(L216)W, W(M130)F	33.0
S124	+F(L216)W, W(M127)F, W(M129)F	20.4
S125	+F(L216)W, W(M129)F, W(M130)F	16.9
S126	+F(L216)W, W(M127)F, W(M130)F	34.0
S127	+F(L216)W, W(M127)F, W(M129)F, W(M130)F	19.0
S155	+F(L216)W, W(M148)F	36.2
S156	+F(L216)W, F(M150)W	20.6
S157	+F(L216)W, W(M148)F, F(M150)W	24.1
S162	+F(M150)W	n.d.
S163	+W(M148)F, F(M150)W	20.9
S205	+L(L185)W, F(L216)W	42.3
S207	+L(L185)W, F(L216)W, W(M148)F	38.6
S208	+L(L185)W, F(L216)W, F(M150)W	29.2
S209	+L(L185)W, F(L216)W, W(M148)F, F(M150)W	30.9
S218	+L(L193)S, F(L216)W	42.1
S222	+L(L185)W, L(L193)S, F(L216)W	39.1
	**Substitutions added to the YFHV background**	
S152	+F(L216)W, W(M148)F	8.1
S153	+F(L216)W, I(M150)W	3.6
S154	+F(L216)W, W(M148)F, I(M150)W	3.8
S158	+ W(M148)F	9.5
S159	+ F(M150)W	4.8
S160	+ W(M148)F, F(M150)W	5.1
S210	+L(L185)W	22.9

Supplementary Figure 5 | Expression yields and $P^+Q_B^-$ yields of RCs carrying combinations of substitutions. Designations of mutant strains and the substitutions they carry are detailed in Supplementary Tables 4 and 5. **a,** Expression yields for RCs carrying combinations of substitutions, determined as described in Methods; **b,** Yields of B-side formation of $P^+Q_B^-$ in RCs carrying combinations of Trp substitutions (blue, YEFHV background; green, YFHV background) or Trp scan substitutions (cyan, YEFHV background; (mean ± SD, n = 3 biologically independent experiments); **c,** Yields of $P^+Q_B^-$ in RCs carrying the ThrL193 and/or TrpL216 substitutions in combination with other substitutions, constructed in YEFHV (blue) or YFHV (green) backgrounds (mean ± SD, n = 3 biologically independent experiments); **d,** Effects on the yields of B-side formation of $P^+Q_B^-$ in RCs that combine the smaller SerL193 with TrpL216 as compared to RCs that carry the larger, branched ThrL193 in combination with TrpL216. Substitution of the native LeuL185 (present in S103/S217/S218) by Arg (S189/S219/S220) or Trp (S205/S221/S222) also influences the overall yield of $P^+Q_B^-$ (mean ± SD, n = 3 biologically independent experiments).

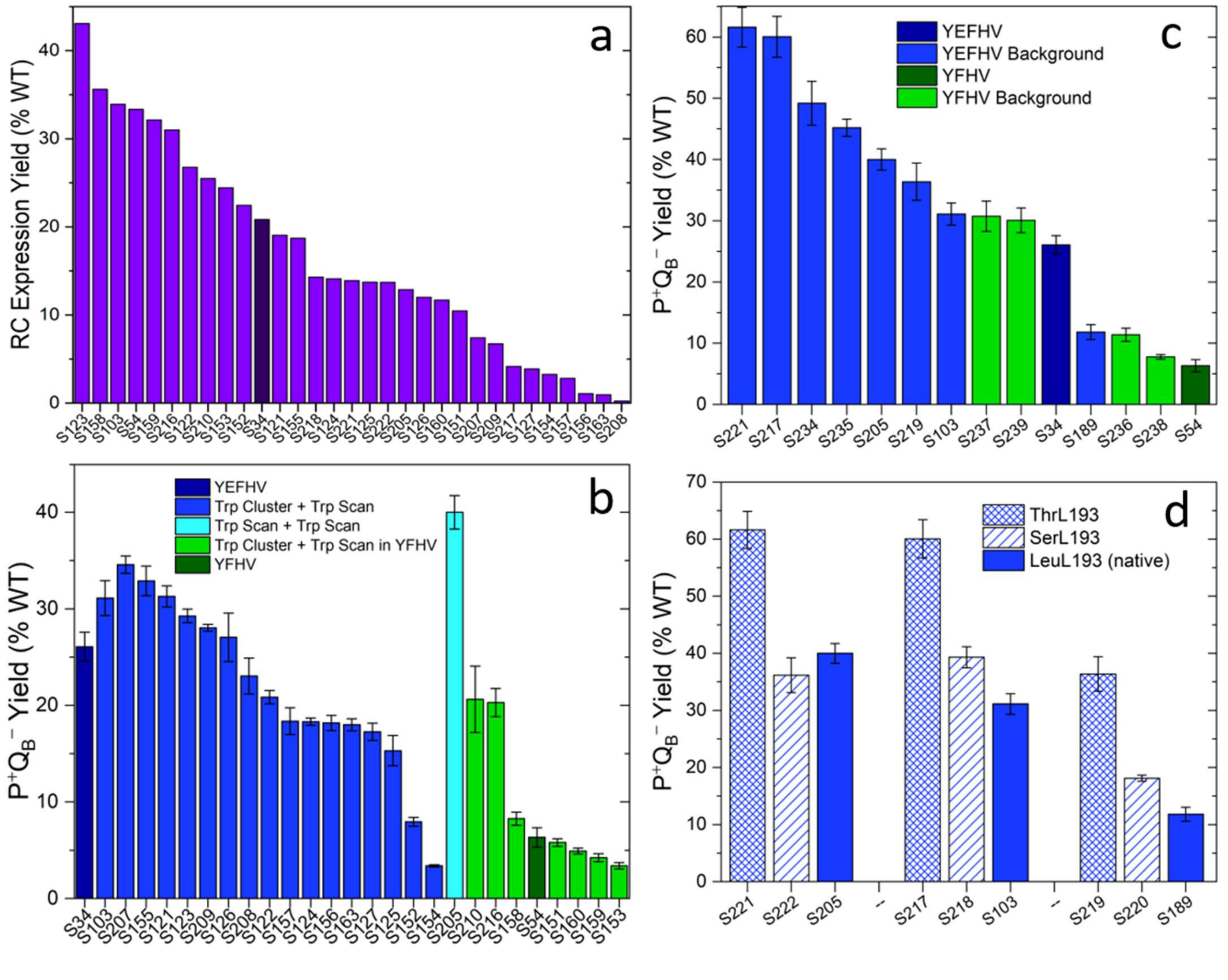

Supplementary Figure 6 | Ground State Spectra.
Spectral features of RCs that combine Trp-cluster and Trp-scan substitutions (**a,b**,or **c,d**) or multiple Trp-cluster/Trp-scan designs with the ThrL193 substitution (**e,f**). The designations of mutant strains and the substitutions they carry are detailed in Supplementary Table 5. **a,c,e,** Ground state spectra of combination mutants, from 250-1000 nm (**a,c,e**) and the Q_y spectral region (700-950 nm; **b,d,f**). Spectra were normalized to a constant value for P_{max} near ~865 nm and were offset by a defined value for overlay.

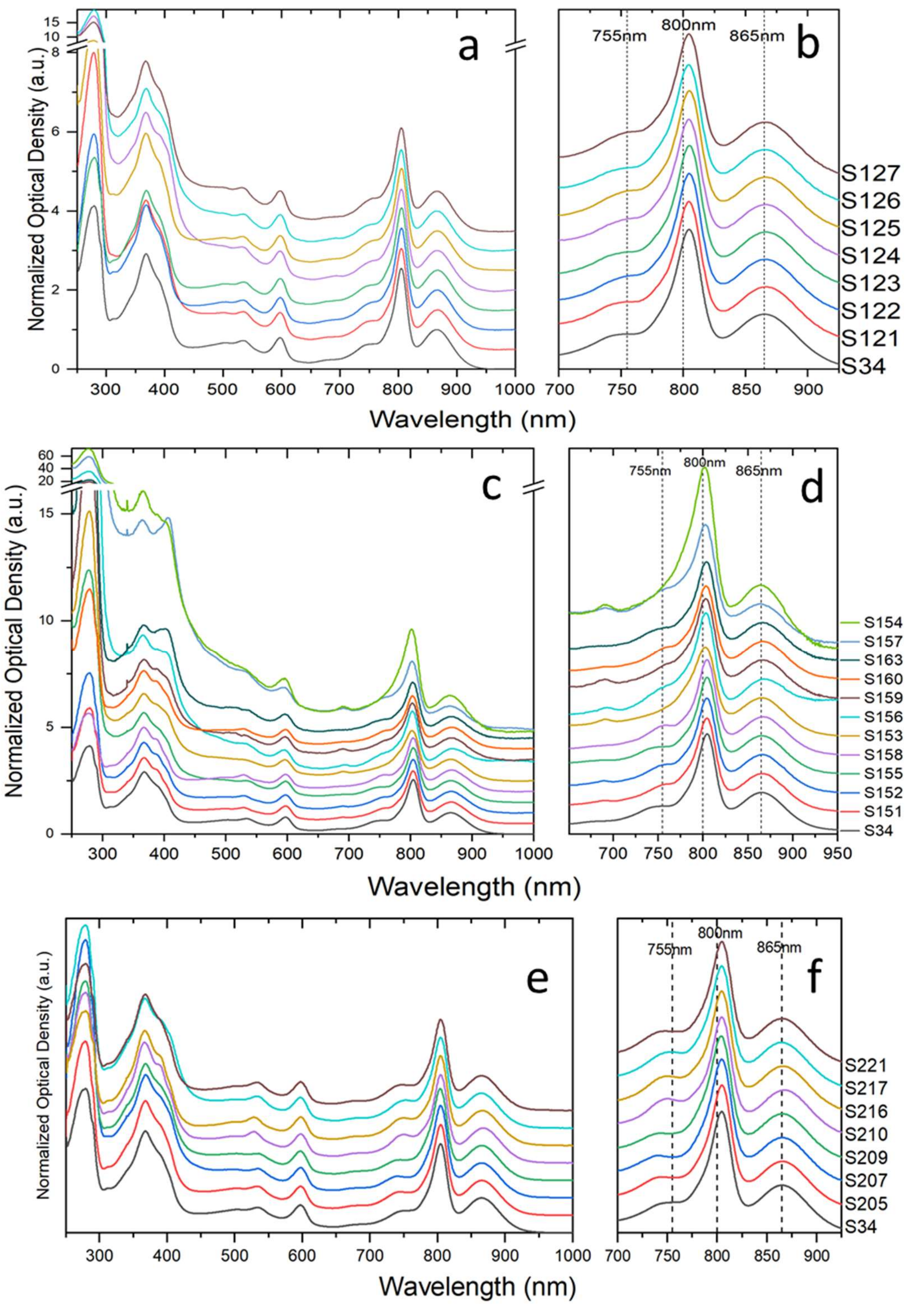

Supplementary Figure 7 | Kinetics of $P^+Q_B^-$ charge recombination in mutant RCs isolated from cultures harvested at low or high cell densities. Examples from native and selected mutant RCs of this study illustrating dependence of kinetics of $P^+Q_B^-$ CR on cell density at time of harvest. The decay of $P^+Q_B^-$ to the ground state was monitored at 850 nm using the millisecond assay, as described in the Methods, and normalized to the maximum of the bleach for direct comparison. CR kinetics for both the WT (**a**; indirect route) and the mutant RCs (**b-d**; direct route) are complex and cannot be described by a single exponential function. The level of heterogeneity in the CR kinetics increases in RCs that are purified from cultures harvested at a higher cell density as compared to data from those purified from lower cell density cultures. Note that $P^+Q_B^-$ in WT RCs is formed from $P^+Q_A^-$ via A-side ET, and CR occurs via an indirect route that involves repopulation of the $P^+Q_A^-$ state. In mutant RCs (**b-d**), $P^+Q_B^-$ is formed via the exclusive activity of the B-path cofactors. At sufficiently long times, all curves converge to $\Delta A = 0$.

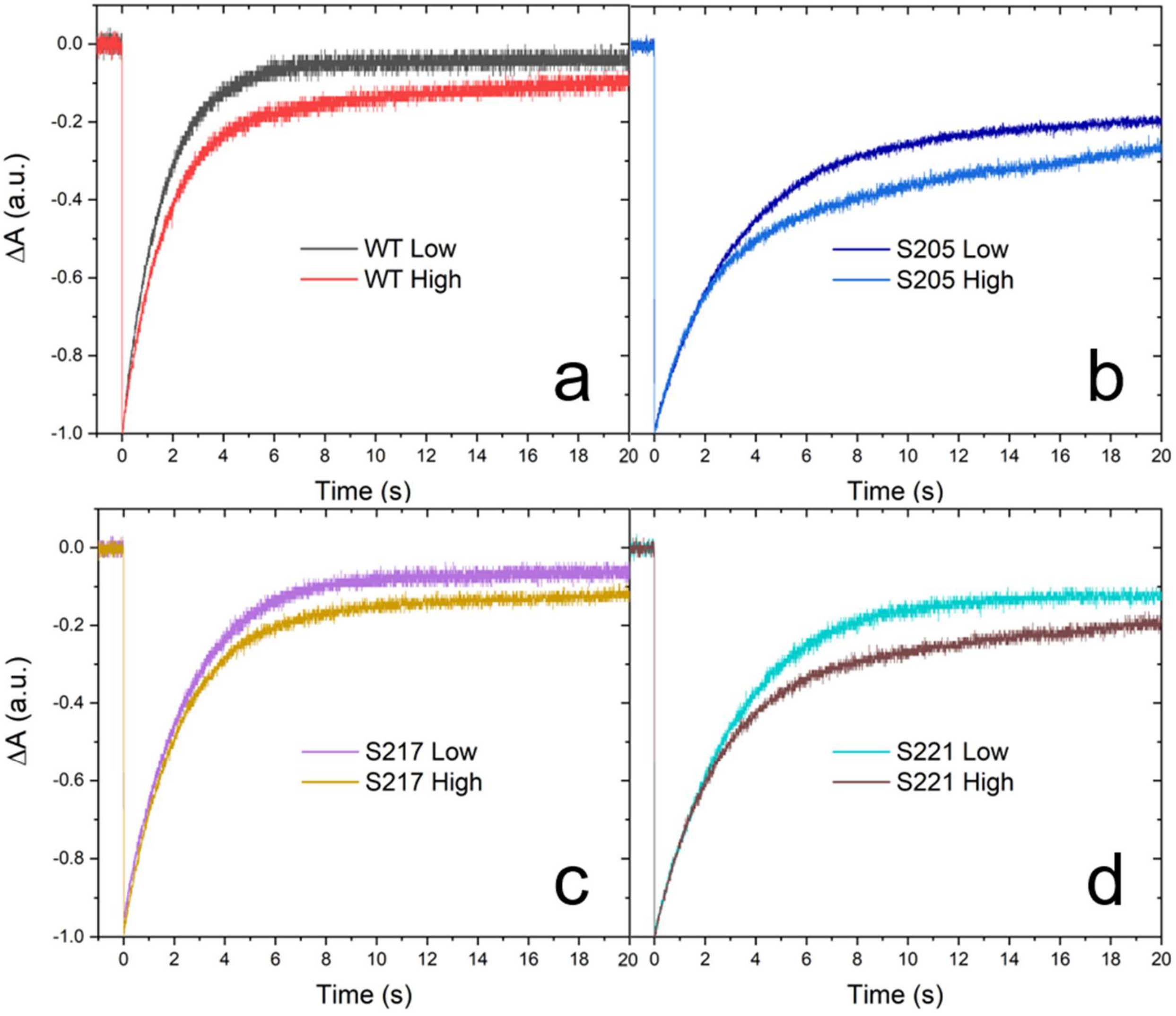

Supplement: Supplementary file 1 — Transparent Peer Review file [file 42004_2025_1460_MOESM1_ESM.pdf]
